# Application of plant–soil feedbacks in the selection of crop rotation sequences

**DOI:** 10.1002/eap.2501

**Published:** 2022-02-06

**Authors:** Akihiro Koyama, Teresa Dias, Pedro M. Antunes

**Affiliations:** ^1^ Algoma University Sault Ste. Marie Ontario Canada; ^2^ Department of Forestry Michigan State University East Lansing Michigan USA; ^3^ Centre for Ecology, Evolution and Environmental Changes University of Lisbon Lisbon Portugal

**Keywords:** arbuscular mycorrhizal fungi, crop rotation, legume, nitrogen, nutrient legacies, plant–soil feedback, soil biota

## Abstract

Plant–soil feedback (PSF) can be a major driver of plant performance in communities, and this concept can be used in selecting crop rotation sequences to maximize agricultural yields. Potential benefits of using PSF in this context include nutrient use optimization, pathogen reduction, and enhancement of mutualisms between crops and microbes. Yet the contributions of these combined mechanisms are poorly understood. Here we investigated the relative contributions of these mechanisms using five major crops commonly cultivated in rotation (alfalfa, canola, maize, soybean, and wheat) under controlled conditions. We trained soil by growing each of the five crops in a “training phase,” and then reciprocally planted the five crops in the trained soils in a “feedback phase.” To tease out soil biota from nutrient effects, we established three treatments: “control” (trained unsterilized soil used in the feedback phases), “biota” (sterilized soil in the feedback phase inoculated with soil biota from the control treatment after the training phase), and “nutrient” (sterilized soils in both phases). Plant–soil feedback for each crop was calculated by comparing the total biomass of each crop grown in soils trained by each of the four other crops (i.e., in rotation) against total biomass in self‐trained soil (i.e., monocropping). We found that PSF values varied among crop combinations in all the treatments, but such variation was the greatest in the nutrient treatment. Overall, soil biota feedback tended to be lower, whereas nutrient feedback tended to be greater compared to the unsterilized control soil, suggesting that effects of antagonistic biota outweighed those of beneficial microbes in the biota treatment, and that plants optimized nutrient uptake when the soil microbiome was absent in the nutrient treatment. Furthermore, soils in the nutrient treatment trained by the legume crops (alfalfa and soybean) tended to provide the greatest positive feedback, emphasizing the important legacy of N_2_ fixers in crop rotation. Taken together, our data demonstrate how nutrients and soil biota can be integral to PSFs among crops, and that assessing PSFs under controlled conditions can serve as a basis to determine the most productive crop rotation sequences prior to field testing.

## INTRODUCTION

Repeated planting of the same crop (monocropping) can result in reduced crop yields because of pathogen accumulation, nutrient depletion, and autotoxication (Cesarano et al., 2017; Delogu et al., 2003; Huang et al., 2013). Crop rotations help maintain long‐term crop yields without heavily depending on synthetic chemicals such as fertilizers, pesticides, and herbicides (Wezel et al., 2014). Given the projected increasing global demand for food, limited land availability for food production, and negative environmental impacts caused by conventional intensive agricultural practices, developing systematic methods to manage crop rotations effectively is paramount (Dias et al., 2015).

Despite the critical importance of crop rotation in sustainable agriculture, there are no established practices to assess the reciprocal effects of multiple crops concurrently (Dury et al., 2012). However, for the past three decades, community ecologists have investigated and developed a framework around the wide suite of positive and negative effects that plants can impose on their rhizosphere and their potential consequences on plant fitness and community structure. Such a line of inquiry is best demonstrated by plant–soil feedback (PSF) experiments in natural systems (e.g., Bever, 1994; van der Putten et al., 2013). Plant–soil feedback can be defined as an ecological process in that a plant leaves biotic and abiotic soil legacies that in turn affect the performance of subsequent plants (Bever, 1994; Brinkman et al., 2010). As crop yields can vary depending on sequence combinations for a set of multiple crops (Benitez et al., 2017), we expect that PSF has the potential to serve as a valuable tool in the process to determine the most favorable crop rotation sequences so that yields can be maximized while maintaining sustainability goals (Stoate et al., 2001).

The application of PSF to determine best crop rotation sequences depends on understanding how crop performance follows soil legacies of previous crops. There are four major factors that drive PSFs: antagonistic biotas (e.g., pathogens), mutualists (e.g., symbiotic nitrogen‐fixing bacteria, mycorrhizal fungi), nutrients, and secondary chemicals (Mariotte et al., 2018; Smith‐Ramesh & Reynolds, 2017). Antagonistic biotas tend to accumulate with monocropping and when phylogenetically close crops are consecutively cultivated, resulting in reduced yields over time (Bever et al., 1997; Miller & Menalled, 2015). Such antagonistic biotas include *Leptosphaeria maculans* and *Leptosphaeria biglobosa* fungi, which cause blackleg disease in canola (*Brassica napus*; Harker et al., 2015), parasitic nematodes in maize (*Zea mays*) and soybean (*Glycine max*) (Grabau & Chen, 2016a, 2016b), and *Gaeumannomyces graminis* (take‐all fungus) in wheat (*Triticum aestivum*) (Cook, 1981). Crop rotations can break such disease cycles (Cheatham et al., 2009; Krupinsky et al., 2002; Peters et al., 2003) and alleviate yield losses due to monocropping.

One of the most important mutualisms in soils involves arbuscular mycorrhizal fungi (AMF), which associate with most major crops (Jansa et al., 2006). The most well‐known benefit for host plants provided by AMF is enhanced uptake of phosphorus (P) (Elbon & Whalen, 2015; Kothari et al., 1991; Smith et al., 2003). Phosphorus is often a limiting nutrient for crop yield (Balemi & Negisho, 2012; Elser, 2012), partly because most of the naturally occurring P in soils is not readily available to plants (Bünemann, 2015). In addition, AMF can provide a suite of other benefits to host plants, including improved acquisition of other macro‐ and micronutrients (Dias et al., 2018), plant pathogen suppression (Veresoglou & Rillig, 2012), increased drought tolerance (Al‐Karaki et al., 2004; Sylvia et al., 1993), alleviation of salt stress (Evelin et al., 2009), and heavy metal tolerance (Gamalero et al., 2009). As a result, stronger crop–AMF associations can significantly stimulate productivity (Treseder, 2013). Community structure of AMF can vary among crops (Bainard et al., 2014) and crop rotations can alter AMF abundance (Tian et al., 2019) and community structure (Zhang et al., 2020). Thus, crop rotation sequences may influence crop yields through AMF abundance and community structure legacies imparted by preceding crops, with some AMF species/isolates being able to overrule the soil's legacy from monocropping (Dias et al., 2018).

Limiting nutrients, such as nitrogen (N), can be depleted from soils in monocropping, resulting in reduced yields over time (Cassman et al., 1995; De Datta et al., 1988). Nitrogen is often the most limiting nutrient in agricultural systems, and synthetic N fertilizer is applied to sustain yields in intensive agriculture (Nkebiwe et al., 2016), which can cause severe environmental issues such as groundwater contamination (Pavlidis & Tsihrintzis, 2018), eutrophication of surface water (Smith & Schindler, 2009), emission of N_2_O from soils (Venterea et al., 2012), and biodiversity loss (e.g., Bobbink et al., 2010). One widely practiced alternative to replenish N to soils is the incorporation of legumes in crop rotations. Legume roots can associate with N_2_‐fixing bacteria (Tautges et al., 2018), which can convert atmospheric N_2_ to bioavailable N (Fustec et al., 2010; Thilakarathna et al., 2016). Commonly used legumes in temperate crop production regions include soybean and alfalfa (*Medicago sativa*) (Kulkarni et al., 2018), which, in rotation, benefit the yields of subsequent crops such as canola (Shrestha et al., 1998), maize (Coombs et al., 2017), and wheat (Kirkegaard et al., 2008).

Some crops synthesize allelopathic chemicals, which can contribute to yield losses under monocropping (Huang et al., 2013). Such crops with autotoxicity include alfalfa (Chon et al., 2002; Miller et al., 1988), canola (Yasumoto et al., 2011), and wheat (Lodhi et al., 1987; Wu et al., 2001, 2007). Crops with autotoxicity can also affect the growth of different plants because of their allelopathic compounds, including that of subsequent crops (e.g., Asaduzzaman et al., 2015; Yasumoto et al., 2011). Allelopathy can influence the structure of rhizosphere biotas, including antagonistic and beneficial microbes, which in turn affect the performance of subsequent crops. For instance, take‐all disease in wheat can be suppressed by allelopathic compounds of canola (Angus et al., 1994). Thus, wheat can benefit when grown after canola instead of monocropping. On the other hand, allelopathy can suppress beneficial soil biotas, resulting in negative effects on subsequent crops. For instance, AM fungal colonization in maize roots can be reduced when planted after canola (Koide & Peoples, 2012), in part because of allelopathy and the nonmycorrhizal nature of canola (Hirrel et al., 1978; Mozafar et al., 2000). Thus, indirect effects of allelopathy may turn out neutral, positive, and negative effects through rhizosphere biotic interactions.

**FIGURE 1:**
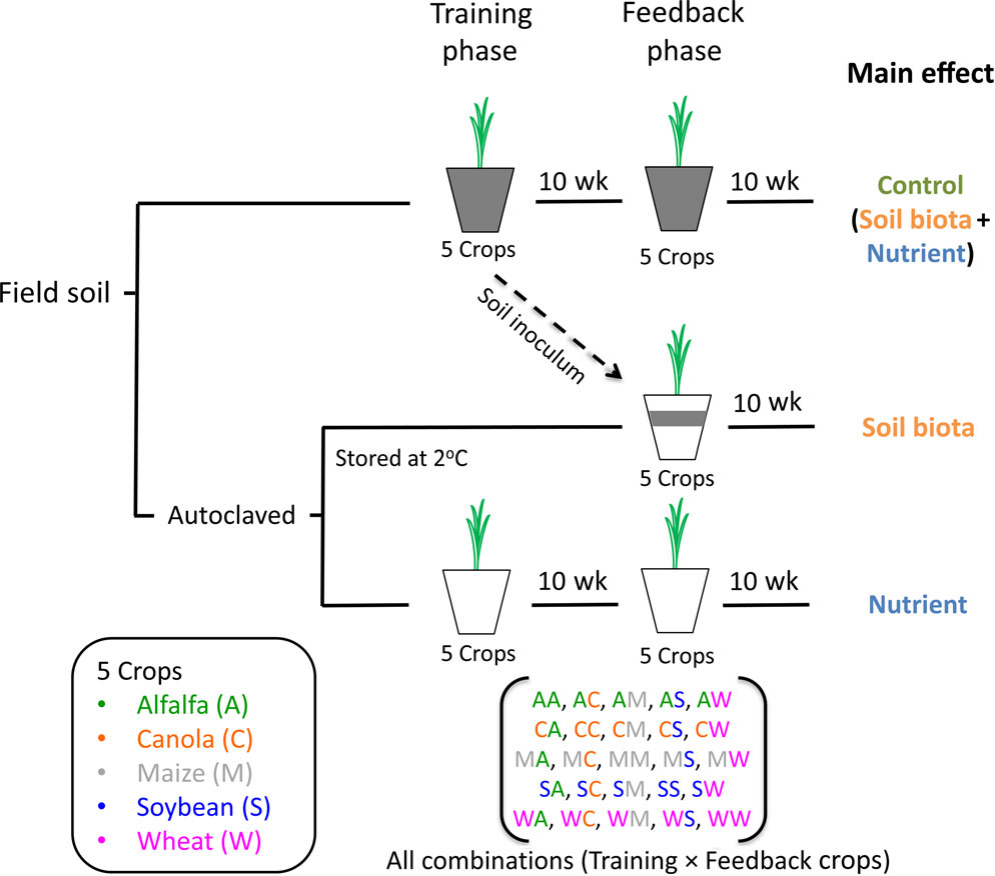
Experimental design to examine biotic and abiotic soil feedback in crop rotations. In the “training phase,” live and sterilized soils were conditioned by the five heterospecific crops for 10 weeks. In the subsequent “feedback phase,” each crop was either grown on self‐conditioned soil or in soil conditioned by each of the other crops. This design resulted in “control,” “biota,” and “nutrient” treatments. Adapted and modified from Dias et al. (2015)

Although we have some knowledge that crop yield can depend on the identity of the preceding crop, most of the available insight comes from data on aboveground pests and soil nutrient availability. Even though PSF has been shown to be important for plant growth and fitness, only a handful of studies have explored how it can influence the performance of agricultural crops in rotation (Benitez et al., 2017; Menalled et al., 2020a, 2020b; Miller & Menalled, 2015). In this study, we evaluated PSFs for five crops (alfalfa, canola, maize, soybean, and wheat) that are commonly grown at a global scale (Leff et al., 2004), including in‐crop rotations. We aimed to tease apart the contributions of soil biotas and nutrients in PSF and elucidate potential mechanisms that determine crop performance. To achieve these goals, we conducted an experiment consisting of training and feedback phases (Figure 1). In the training phase, live and sterilized field soils were conditioned by each of the five crops. In the feedback phase, the conditioned soils were used to set up three treatments, including “control” (trained unsterilized soil used in the feedback phases), “biota” (sterilized soils inoculated with a small portion of live soils trained by each crop), and “nutrient” (sterilized soils trained by each crop) (Figure 1). In each treatment, we had a total of 25 combinations of the five crops in both phases (i.e., 5 × 5) replicated eight times (Figure 1). We measured plant biomass at the end of the feedback phase to calculate PSFs by comparing total biomass of each crop grown in soils trained by each of the four other crops (i.e., in rotation) against biomass in self‐trained soil (i.e., monocropping) using total biomass (Brinkman et al., 2010; Petermann et al., 2008). We hypothesized that (1) PSF varies among all crops and soil treatments; (2) control feedback values can be predicted using biotic and nutrient feedback values. In the process of testing the first hypothesis, we assessed correlations between four plant characteristics (shoot N content, root lesion frequency, and arbuscule and vesicle colonization rates) and biomass to gain insights into nutrient and soil biota influence on plant performance. In testing the second hypothesis, we specifically quantified the relative contributions of soil nutrient and biotic legacies of each crop on any other crop. Our goal with this approach is to understand the role of beneficial and detrimental soil organisms and soil nutrient legacies on crop productivity in crop rotations.

## MATERIALS AND METHODS

### Experimental design and plant growing conditions

This experiment was designed to investigate PSFs using five crops and to tease out soil biota and nutrient components of the feedbacks (Figure 1) (Dias et al., 2015). To achieve these objectives, three treatments (control, biota, and nutrient) were set up as a pot experiment (Figure 1). The first phase of the experiment was used to condition soils by each of the five crops (training phase), and the following phase was used to investigate the growth of each crop in soil trained by each of the five crops (feedback phase; Figure 1). Accordingly, the training phase resulted in a total of (1) 200 pots (i.e., five training crops × five feedback crops × eight replications) for each treatment (control and nutrient) and a total of 400 in the training phase; and (2) 600 in the feedback phase (control, biota, and nutrient). The experiment was conducted in a glasshouse facility at the Ontario Forestry Research Institute in Sault Ste. Marie, Ontario, Canada.

Five heterospecific crop cultivars selected in this study are commonly cultivated in the Algoma region, Ontario, Canada; alfalfa (*M. sativa*, common alfalfa, locally cultivated undetermined variety), canola (*B. napus*—L150, Bayer), maize (*Z. mays*—Pioneer 39B90, Roundup Ready), soybean (*G. max*—900Y81, Roundup Ready), and wheat (*Triticum* spp., Sable, JGL Inc.).

Topsoil (20‐cm depth) was collected from an agricultural field (Algoma Community Pasture, 46°17′01.7″ N 83°31′33.0″ W) in September 2013. The soil in the area is categorized as Orthic Humic Gleysols (Griffith et al., 1984). Soil organic matter content was 6.0% (±0.1 SD) and soil pH in water (Thomas, 1996) was 6.0 (±0.1 SD) (Agriculture and Food Laboratory, University of Guelph, Guelph, Ontario, Canada). The soil was first sieved using a 4‐mm mesh screen and two‐thirds was sterilized in an autoclave (121°C for 90 min, repeated twice and then left untouched for 1 week) and used for the biota and nutrient treatments whereas the rest of the live soil was used for the control treatment without sterilization (Figure 1). Each of the live and sterilized soils was mixed with sterilized (autoclaved at 121°C for 90 min, repeated twice) noncalcareous granitic sand (Hutcheson Sand and Mixes, Huntsville, Ontario, Canada) and Turface (a montmorillonite clay, Turface Athletics MVP, Profile Products LLC, Buffalo Grove, IL, USA) (soil:sand:Turface 7.5:1:1 in volume). Using this mixture slightly changes the soil texture, which facilitates handling plant roots in downstream processes (e.g., washing roots to detach soil without losing root materials for accurate biomass quantification). Half of the mixed sterilized soil was stored in sealed plastic containers at 2°C for the biota treatment in the feedback phase, and the rest of the mixed sterilized soil and live soil were used for the nutrient and control treatments, respectively, in the training phase, where the soils were conditioned by each of the five crops.

In both training and feedback phases, plants were grown in 1.65‐L pots (“Short‐One” model, 10 × 23 cm, Stuewe and Sons, Corvallis, Oregon, USA). Pots were cleaned with household bleach diluted by 10‐fold followed by rinsing with tap water. To set up the “control” treatment in the training phase, 1.8 kg (fresh weight) of live mixed soil were placed in each of the 200 pots. In a same manner, 1.8 kg of sterilized mixed soil was placed in each of 200 pots to set up the nutrient treatment in the training phase. To reduce any potential microbial contamination from agricultural soil, the benches and floor in the glasshouse facility were cleaned with a diluted household bleach solution, followed by rinsing with tap water, at the onset of the training and feedback phases.

Seeds of the five crops were first surface‐sterilized by immersion for 5 min in a 10% household bleach solution, followed by three rinses of autoclaved deionized water. The seeds were germinated on moisturized autoclaved vermiculite (in the training phase) and paper towels (in the feedback phase) in trays at 26°C in a growth chamber. We note that germination rates can vary among crops because of PSF (e.g., Menalled et al., 2020b). In this study, however, our goal was to determine the role of PSF on plant performance after germination.

### Soil training phase

Because soybean had never been grown in the field where the soil was collected, a commercial liquid inoculum containing symbiotic N_2_‐fixing bacteria (*Bradyrhizobium japonicum*, Nodulator, BASF, Mississauga, Ontario, Canada) was added to each pot in the control soils at the beginning of the training phase; each pot received 5 ml of “Nodulator” diluted 31× with double deionized water. This relatively high concentration (i.e., 166× greater than the manufacturer's recommendation), was chosen to ensure the nodulation of soybean roots without any known detriment to other soil biota. Autoclaved inoculum was added to each pot in the nutrient treatment at the beginning of the training phase.

Four seedlings of each crop were transplanted into a pot filled with mixed soil to initiate the training phase. After 3 weeks, plants were thinned to one plant per pot. Watering regime depended on plant growth and changed during the training phase; deionized water was added to each pot twice a week so that gravimetric water content was maintained between 40% and 60% for the first 7 weeks, and at field capacity for the remaining period. The plant growth conditions were set as 15 h at 20°C for daytime, and 9 h at 12°C for nighttime, based on the average temperature of the growing season of the Algoma region, Ontario, Canada. Daytime light conditions ranged from approximately 100 to 400 μmol m^−2^ s^−1^ of photosynthetically active radiation with supplemental light from sodium‐vapor lamps. Plants were grown for 10 weeks in the training phase between October 2012 and January 2013. Pot locations in the glasshouse were completely randomized at the beginning of the training phase, and once in the seventh week.

At the end of the training phase, shoots were harvested by cutting stems at the soil surface. Roots were left intact in soils. Each shoot was placed in a paper bag, dried at 60°C for 48 h in an oven, and weighed. From each pot in the control treatment, 50 g of fresh soil was removed to be used as an inoculum for the biota treatment in the feedback phase (Figure 1). The 50‐g soil was placed in a sealable plastic bag and stored at 4°C until when the feedback phase was initiated. Removed soil from each pot was replaced with 50 g of sterilized soil (fresh weight) stored at 2°C. To make the treatments comparable, the same process was applied to each pot in the nutrient treatment at the end of the training phase.

In between the training and feedback phase (12 days), soils were left in the greenhouse chamber for 1 week at the previously described conditions. Pots were watered prior to the shoot harvest. As such, in absence of transpiration, any potential water loss in between phases was negligible. Soils were then maintained at 12°C and ambient light conditions in the greenhouse to maintain soil biota community until planting.

### Feedback phase

The biota treatment was implemented in this phase and consisted of placing 1,750 g of the sterilized mixed soil (which had been stored at 2°C) in each of 200 clean pots. Each pot in the biota treatment further received 50 g of soil inoculum (corresponding to 2.8% by weight), which had been collected from an individual control pot at the end of the training phase. This inoculation method using a small quantity of biota soil has been verified (Brinkman et al., 2010) and widely used in PSF experiments (e.g., Kempel et al., 2018; Maron et al., 2014; McHaffie & Maherali, 2020).

The feedback phase consisted of 25 factorial combinations (i.e., five crops in the training phase × five crops in the feedback phase) for each of the control, nutrient, and biota treatments with eight replications per crop × treatment combination. Four seedlings of each crop were transplanted to soil in each pot conditioned in the training phase. Plants in the feedback phase were grown for 10 weeks in the same manner as in the training phase between January and April in 2013. Pot locations were randomized at the beginning of the feedback phase, and every 2 weeks during the 10‐week period. The process of adding *B. japonicum* inoculum was applied to the biota treatment at the beginning of the feedback phase in the same manner as described for the training phase.

At the end of the feedback phase, above‐ and belowground biomass of all the plants was harvested. Shoots and roots were separated at the soil surface. Shoots were dried at 60°C for 3 days to calculate dry shoot biomass. Roots were gently washed in tap water to remove soils, tap‐dried between paper towels, and weighed. For alfalfa and soybean plants, presence or absence of nodules in roots was visually inspected. A fraction of roots from each sample were placed in 50% ethanol to assess colonization of AMF and root lesions (i.e, root herbivory, damage and decay, Schnitzer et al., 2011). The rest of the roots were dried at 60°C for 3 days to calculate total dry root biomass.

Four shoot samples were randomly selected from each set of eight replications to quantify total nitrogen (N) and carbon (C) content. The dried shoot samples were finely ground and homogenized using a Retsch MM400 mixer mill (Retsch GmbH, Haan, Germany), and N and C content of each sample was analyzed using a Flash 2000 Organic Elemental Analyzer (Thermo Fisher Scientific, Bremen, Germany).

To assess AM fungal colonization (McGonigle et al., 1990) and lesions, four root samples were randomly chosen from each set of eight replications for each crop. To quantify AM fungal colonization, roots were first cleared in 10% KOH at 80°C for 30 min in a water bath. After acidification for 15 min in 1% HCl, the roots were stained in 0.05% trypan blue solution (1:1:1 glycerol, lactic acid and deionized water) at 80°C for 30 min, and stored in 50% glycerol. Roots were mounted in 50% glycerol on microscope glass slides (Corning Inc., New York, USA), and arbuscules, vesicles, and hyphae colonization was quantified at ×200 magnification using the intersection method (McGonigle et al., 1990). To quantify lesions, roots cleared in 10% KOH were mounted in 50% glycerol on microscope glass slides, and lesions were counted (Schnitzer et al., 2011).

### Statistical analyses

Using the total biomass data, PSF values were derived using a modified formula from Petermann et al. (2008):

$$\text{Feedback}=\log\left(\frac{\text{Biomass in soil trained}\;\mathrm{by}\;\text{different crop}}{\text{Biomass in self}-\text{trained soil}}\right).$$

Feedback values were calculated by comparing total biomass of each crop grown in soils trained by each of the four other crops against biomass in self‐trained soil via bootstrapping. Bootstrapping is one of the few methods that can estimate the uncertainty associated with log‐response ratios in PSF experiments in a nonblocked design such as that of this study (Bates et al., 2020). Bootstrapping was performed in R (R version 3.5.0; R Development Core Team, 2020) with 999 iterations, and eight resulting feedback values were randomly selected within a 95% confidence interval. These randomly selected values were used for statistical analyses.

Fixed‐effect ANOVAs (Type III in R version 4.0.2, R Core Team, 2020) were used to test if the soil treatments (i.e., control, nutrient, and biota) and the training crops were significant factors in determining individual plant characteristics (e.g., total biomass, shoot N content, root lesion frequency, and colonization rates of arbuscules, vesicles, and hyphae) as well as feedback values for each crop. When necessary, appropriate transformations were made to meet the assumption of normality and to stabilize residual variances. When the three soil treatments were found significant in ANOVAs, Tukey multiple‐comparison analyses were conducted to test differences among the treatments for each crop. For some plant characteristics, only two soil treatments could be compared (e.g., live and autoclaved soils for shoot biomass in the training phase). In these cases, Student's *t* tests were conducted for each crop to test differences between the soil treatments. Pearson correlation coefficients were calculated to assess relationships between plant characteristics (shoot N content, root lesions, AM mycorrhizal colonization) and total biomass using values averaged for each crop by feedback combination.

To assess if feedback values in the control treatment could be predicted by feedback values in the biota and/or nutrient treatments for all the reciprocal combinations of soil treatments and crops, linear fixed‐effect models were employed. Using mean feedback values, four models were constructed with control feedback values as the response variable. Predictive variables of the four models were feedback values of (1) biota treatment only, (2) nutrient treatment only, (3) biota and nutrient treatment (additive), and (4) biota and nutrient treatment and their interaction. The four models were compared using the Akaike information criteria (AIC; Akaike, 1974) to choose the best model.

All the analyses were conducted using R (R version 4.0.2, R Core Team, 2020) in RStudio (2015). When an outlying data point was observed, a Grubbs’ test (Grubbs, 1950) was conducted to determine if they indeed qualified as an outlier at α = 0.05 using the R package “outliers” (Komsta, 2011).

All the raw data and the R codes used for the bootstrapping are available at Dryad (https://doi.org/10.5061/dryad.sqv9s4n2r).

## RESULTS

**FIGURE 2:**
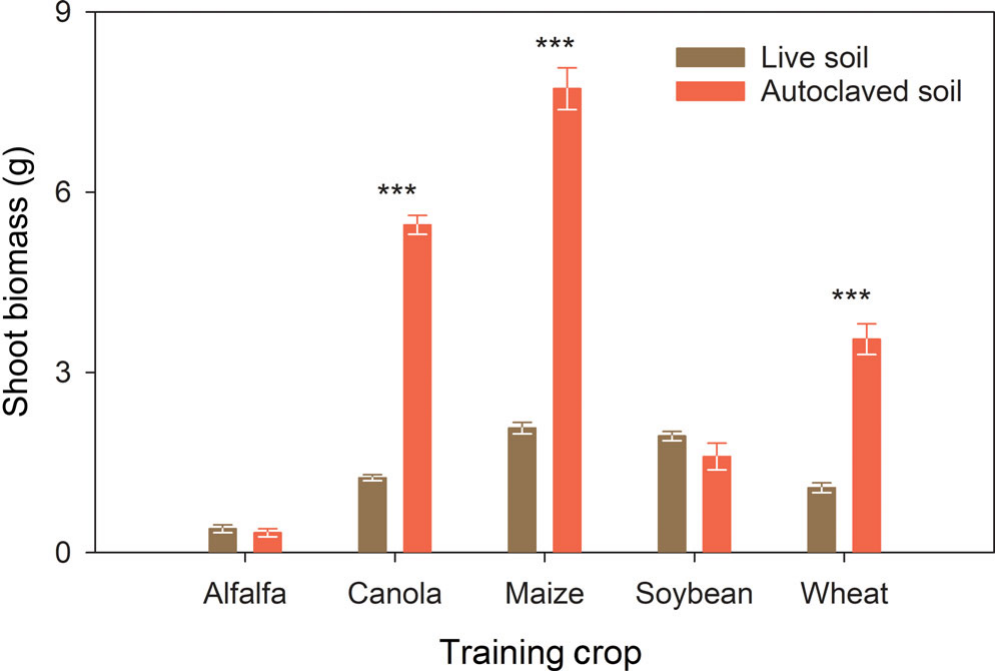
Shoot biomass of the five crops at the end of the training phase in live and autoclaved soils, represented by green and red, respectively. Each bar and the associated error bar represent an average and standard error, respectively. Asterisks above bars show significant differences between the soil treatments for paired samples via Student's *t* tests (***p ≤ 0.001). Absence of asterisks indicates no significant difference between the two soil treatments

In the training phase, differences in shoot biomass between the live and autoclaved soils depended on the crops (Figure 2). This is supported by a soil × crop interaction (*p* < 0.001). Shoot biomass was similar between the two soil treatments for the legumes (i.e., alfalfa and soybean) whereas the three nonlegume crops (i.e., canola, maize, and wheat) had greater biomass in autoclaved than in live soils (Figure 2).

**FIGURE 3:**
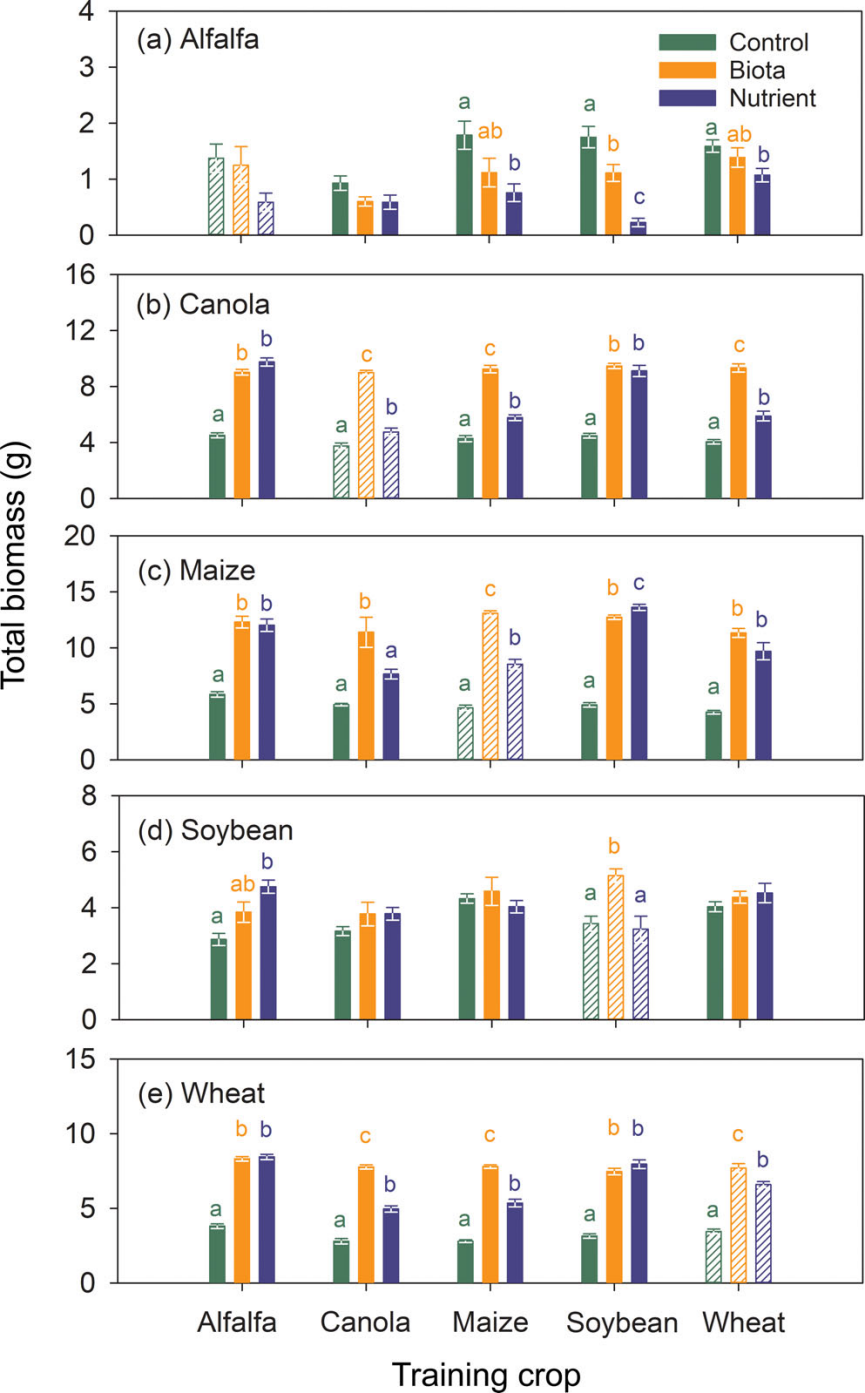
Total biomass of the five crops at the end of the feedback phase under three soil treatments, “control,” “biota,” and “nutrient,” represented by green, orange, and blue, respectively. The hatched bars represent plants grown in continuous monoculture after the training phase (Figure 1). Each bar and the associated error bar represent an average and standard error, respectively. Bars topped by different letters among the three soil treatments for each training × feedback crop combination indicate significant difference at *p* ≤ 0.05 by a Tukey multiple‐comparison test. Bars without letters indicate no significant difference among the three soil treatments

At the end of the feedback phase, total biomass of each of the five crops depended both on the soil treatments and training crops (Figure 3). This was supported by interaction effects for each of the five crops (*p* ≤ 0.013, Table 1). The three nonlegume crops (i.e., canola, maize, and wheat; Figure 3b,c,e) consistently had smaller biomass in the control treatment than in the other two treatments (i.e., biota and nutrient; Appendix S1: Figure S1a,b). In contrast, the two legume crops (i.e., alfalfa and soybean) showed different trends (Figure 3a,d). Alfalfa grew best in the control treatment (Figure 3a; Appendix S1: Figure S1a,b) whereas soybean grew relatively similarly among the three soil treatments and irrespective of training crop (Figure 3d, Appendix S1: Figure S1a,b). No significant correlations were found among soil treatments for each crop's overall total biomass at the end of the feedback phase (*p* > 0.05; Appendix S1: Figure S1), except for canola (*p* = 0.04) and wheat (*p* = 0.08) between the control and biota treatments (Appendix S1: Figure S1a).

**TABLE 1:** ANOVA results to assess effects of the soil treatments and training crops on total biomass of each crop in the feedback phase

Response variable	Feedback crop	Soil treatment	Training crop	Interaction
Total biomass	Alfalfa (Figure 3a)	*F*_2,105_ = 30.01 ***p* < 0.001**	*F*_4,105_ = 5.64 ***p* < 0.001**	*F*_8,105_ = 2.57 ***p* = 0.013**
	Canola (Figure 3b)	*F*_2,105_ = 524.95 ***p* < 0.001**	*F*_4,105_ = 35.61 ***p* < 0.001**	*F*_8,105_ = 21.07 ***p* < 0.001**
	Maize (Figure 3c)	*F*_2,105_ = 273.60 ***p* < 0.001**	*F*_4,105_ = 13.35 ***p* < 0.001**	*F*_8,105_ = 7.24 ***p* < 0.001**
	Soybean (Figure 3d)	*F*_2,105_ = 8.65 ***p* < 0.001**	*F*_4,105_ = 3.455 ***p* < 0.001**	*F*_8,105_ = 4.101 ***p* < 0.001**
	Wheat (Figure 3e)	*F*_2,105_ = 39.00 ***p* < 0.001**	*F*_4,105_ = 8.52 ***p* < 0.001**	*F*_8,105_ = 13.84 ***p* < 0.001**
Feedback values	Alfalfa (Figure 6a)	*F*_2,84_ = 33.06 ***p* < 0.001**	*F*_3,84_ = 104.28 ***p* < 0.001**	*F*_6,84_ = 69.09 ***p* < 0.001**
	Canola (Figure 6b)	*F*_2,84_ = 1234.71 ***p* < 0.001**	*F*_3,84_ = 227.46 ***p* < 0.001**	*F*_6,84_ = 141.23 ***p* < 0.001**
	Maize (Figure 6c)	*F*_2,84_ = 714.26 ***p* < 0.001**	*F*_3,84_ = 379.92 ***p* < 0.001**	*F*_6,84_ = 154.59 ***p* < 0.001**
	Soybean (Figure 6d)	*F*_2,84_ = 315.19 ***p* < 0.001**	*F*_3,84_ = 108.81 ***p* < 0.001**	*F*_6,84_ = 6.31 ***p* < 0.001**
	Wheat (Figure 6e)	*F*_2,84_ = 148.18 ***p* < 0.001**	*F*_3,84_ = 639.53 ***p* < 0.001**	*F*_6,84_ = 190.16 ***p* < 0.001**

*Note*: Total biomass and feedback values are shown in Figures 3 and 6, respectively. Bold *p* values indicate statistical significance (*p* < 0.05).

**FIGURE 4:**
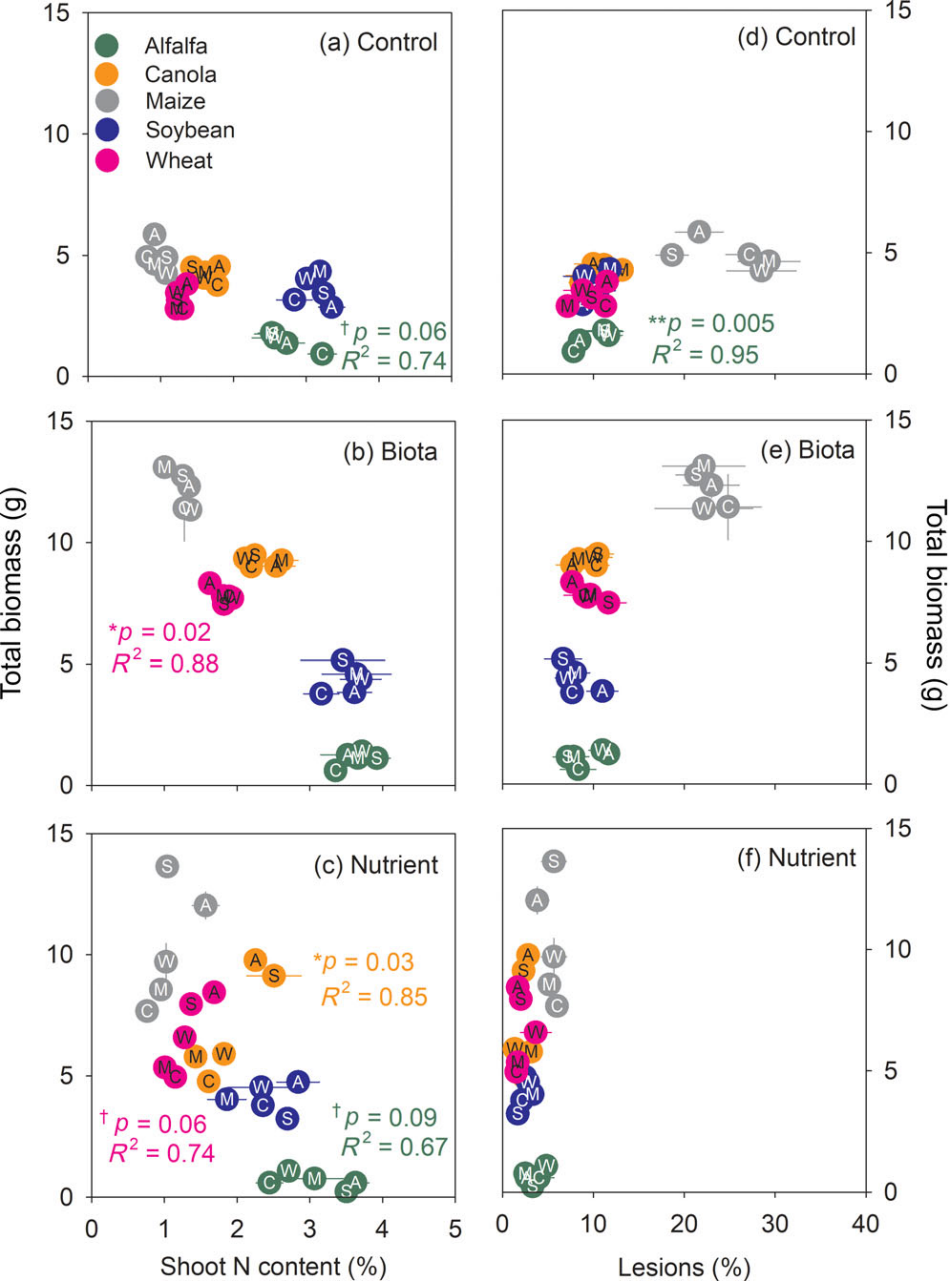
Relationships between plant characteristics (shoot N content and root lesion frequency) and total biomass for each treatment (“control,” “biota,” and “nutrient”). Different colors of the circles represent crops in the feedback phase, and letters in circles represent crops in the training phase: A, C, M, S, and W stand for alfalfa, canola, maize, soybean, and wheat, respectively. Each symbol and the associated error bars represent average and standard errors, respectively (*N* = 8). *p* values and *R* ^2^ show results of Pearson correlation analyses for corresponding crops in the same colors, and associated symbols indicate statistical significance; ***<0.01, **<0.05, †<0.10. Crops without *p* values or *R* ^2^ had no significant correlations between x‐axis values and total biomass

**FIGURE 5:**
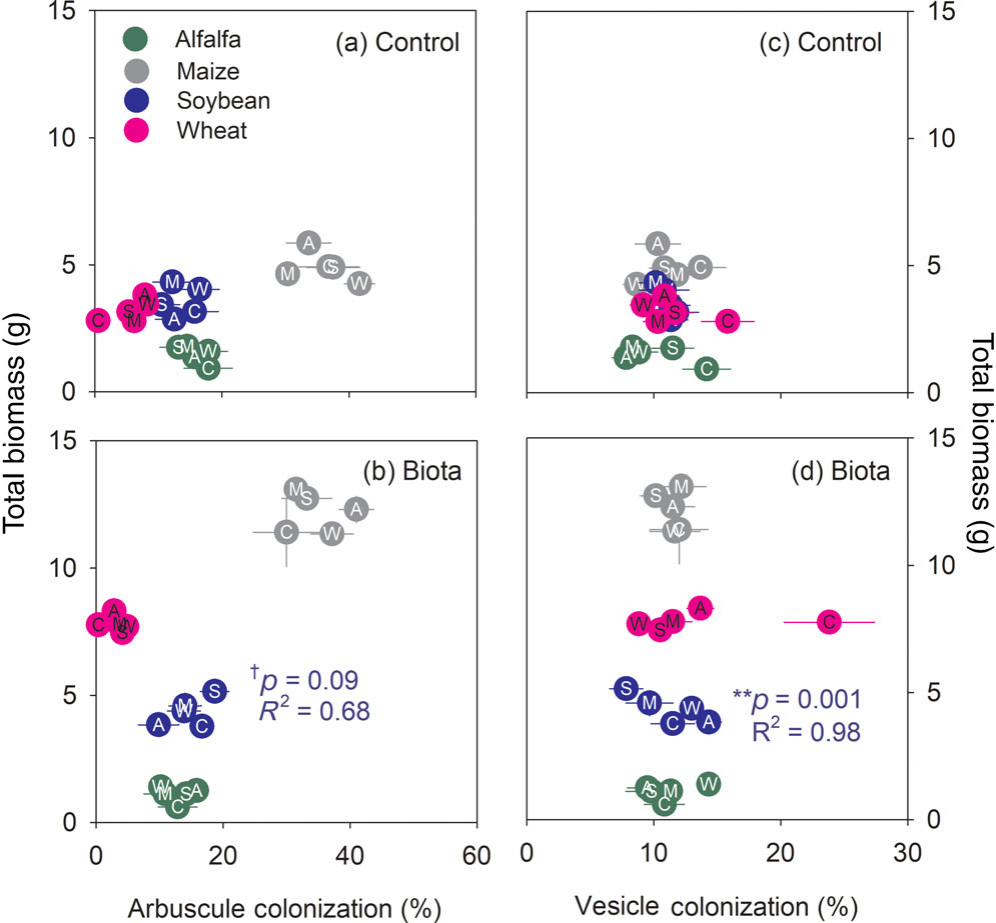
Relationships between arbuscular mycorrhizal colonization (arbuscular and vesicular colonization) and total biomass for each treatment (“control” and “biota”). Different colors of the circles represent crops in the “feedback phase,” and letters represent crops in the “training phase”: A, C, M, S, and W stand for alfalfa, canola, maize, soybean, and wheat, respectively. Each symbol and the associated error bars represent an average and standard errors, respectively (*N* = 8). No arbuscules or vesicles were found in nonmycorrhizal canola or in any crops in the “nutrient” treatment. *p* values associated with symbols, and *R* ^2^ indicate statistical significance from Pearson correlation analyses for soybean; **< 0.01, †< 0.10. Crops without *p* values or *R* ^2^ had no significant correlations between x‐axis values and total biomass

Correlations between total plant biomass and four plant characteristics were assessed for each soil treatment (Figures 4 and 5). The plant characteristics were shoot N content (Figure 4a–c; Appendix S1: Figure S2, Table S1), root lesions (Figure 4d–f; Appendix S1: Figure S3, Table S1) and rate of arbuscular (Figure 5a,b; Appendix S1: Table S1, Figure S4), vesicular (Figure 5c,d; Appendix S1: Table S1, Figure S5) and hyphal root colonization (Table S2, Figure S6). In the control and biota soil treatments, shoot N content and total biomass were negatively correlated for alfalfa (*p* = 0.06; Figure 4a) and wheat (*p* = 0.02; Figure 4b), respectively. In the nutrient soil treatment, shoot N content was a significant predictor for total biomass in three crops (Figure 4c); shoot N content and total biomass were positively correlated for canola and wheat (*p* = 0.03 and *p* = 0.06, respectively; Figure 4c) but negatively correlated for alfalfa (*p* = 0.09; Figure 4c). For canola and wheat, the positive correlations were driven by the high shoot N content in plants grown in soils previously trained by the two legumes (i.e., alfalfa and soybean; Figure 4c). For maize, legume‐trained soils did not notably increase shoot N concentration, but rather increased plant biomass (Figure 4c). No clear correlation between the frequency of root lesions and total biomass was found across the three soil treatments for most crops at the end of the feedback phase. The exception was alfalfa in the control soil where the correlation was positive (*p* = 0.005; Figure 4d).

Arbuscular colonization was not a significant predictor of total biomass for any crop in the control soils (*p* ≥ 0.21; Figure 5a). However, a marginally significant positive correlation between arbuscular colonization rates and total biomass was detected for soybean in the biota soils (*p* = 0.09; Figure 5b). Vesicular colonization was not a predictor of total biomass in the control soils (Figure 5c). However, in the biota soils, there was a negative correlation between vesicular colonization and total soybean biomass (*p* = 0.001; Figure 5d). Hyphal colonization (Appendix S1: Figure S6) was not clearly correlated with total biomass in any crop, irrespective of soil treatment combination at the end of the feedback phase (Appendix S1: Figure S7), except for a negative correlation for soybean in the nutrient soil treatment (*p* = 0.02; Appendix S1: Figure S7c).

**FIGURE 6:**
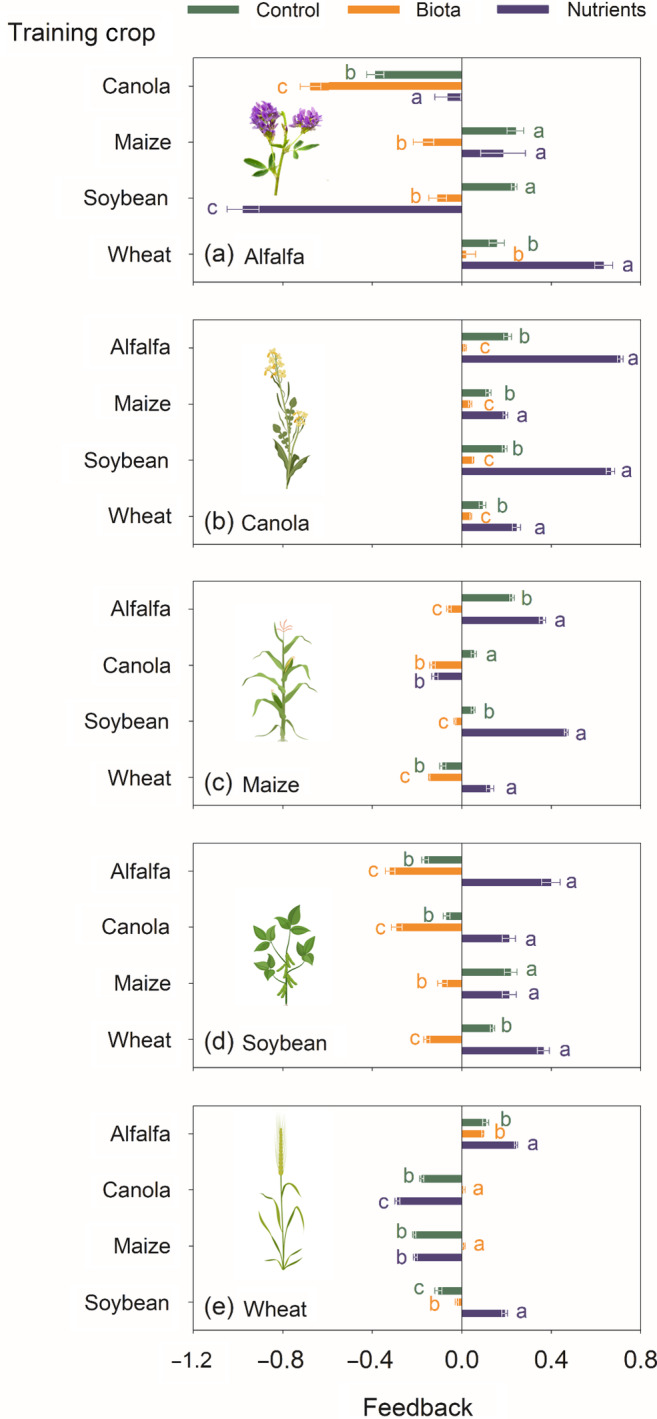
Soil feedback in three soil treatments calculated by comparing the total biomass of each crop grown in soils trained by the four other crops against biomass in monocropping after the training phase via bootstrap. Taking alfalfa as an example, (a) shows the growth of alfalfa after each of the other crops relative to monocropping (i.e., soil trained by alfalfa). Each bar and the associated error bar represent an average and standard error, respectively. Bars associated with different letters among the three soil treatments for each training × feedback crop combination indicate significant difference at *p* ≤ 0.05 by a Tukey multiple‐comparison test

To test the first hypothesis (PSF varies among all crops and soil treatments), we analyzed effects of the training crops and soil treatments on PSF values. Plant–soil feedback for total plant biomass depended on both the soil treatment and identity of the preceding crop (i.e., training crop) (Figure 6). This was supported by consistent soil treatment × crop interaction effects across crops at the end of the feedback phase (i.e., feedback crops) (*p* < 0.001; Table 1). Each soil treatment had varying degrees of both positive and negative feedback; mean plant–soil feedbacks varied from −0.39 (alfalfa in canola‐trained soils) to 0.24 (alfalfa in maize‐trained soils) in the control soils, from −0.67 (alfalfa in canola‐trained soils) to 0.09 (wheat in alfalfa‐trained soils) in the biota soils, and from −0.98 (alfalfa in soybean‐trained soils) to 0.71 (canola in alfalfa‐trained soils) in the nutrient soils (Figure 6).

Of all the five feedback crops, alfalfa had the most varying feedback across the three soil treatments, especially in the nutrient soils, ranging from −0.98 (in soybean‐trained soils) to 0.63 (in wheat‐trained soils) (Figure 6a; Appendix S1: Figure S8a). In contrast, canola showed the most consistent feedback outcomes, generally experiencing neutral or positive responses in soil trained by the other four crops relative to its own trained soil (Figure 6b; Appendix S1: Figure S8b). The most positive biomass responses for this crop were found in the nutrient soils, followed by the control and biota soils (Figure 6b; Appendix S1: Figure S8b). Soybean also experienced consistent feedback effects where it benefited from growing in rotation for nutrients but not for biota (Figure 6d). Responses for this crop varied depending on the training crop in the control soils (Figure 6d). Feedback responses for maize were relatively small in the control and biota soils, except a significant positive response after alfalfa in the control treatment (Figure 6c). Maize showed positive responses to all the other training crops in the nutrient treatment, except for canola (Figure 6c). Wheat had relatively variable feedback, especially in the nutrient treatment, but it responded positively or negatively when either of the two other preceding crops were legumes or not, respectively (Figure 6e).

Among the five training crops, alfalfa and soybean consistently benefited the other crops in the nutrient treatment (Appendix S1: Figures S8f,i, S9a,d) likely via a nutrient legacy with more N from rhizodeposition (Figure 4c). However, the effect of soybean on alfalfa was the most negative in the experiment (Appendix S1: Figure S9d). Wheat consistently benefited all of the other crops in the nutrient treatment (Appendix S1: Figures S8j, S9e). Canola left the most negative plant–soil legacy to the other crops across the three treatments in terms of biomass effects with a few exceptions (Appendix S1: Figures S8g, S9b). As a training crop maize was characterized by having relatively small effects across the three soil treatments (Appendix S1: Figures S8h, S9c).

**FIGURE 7:**
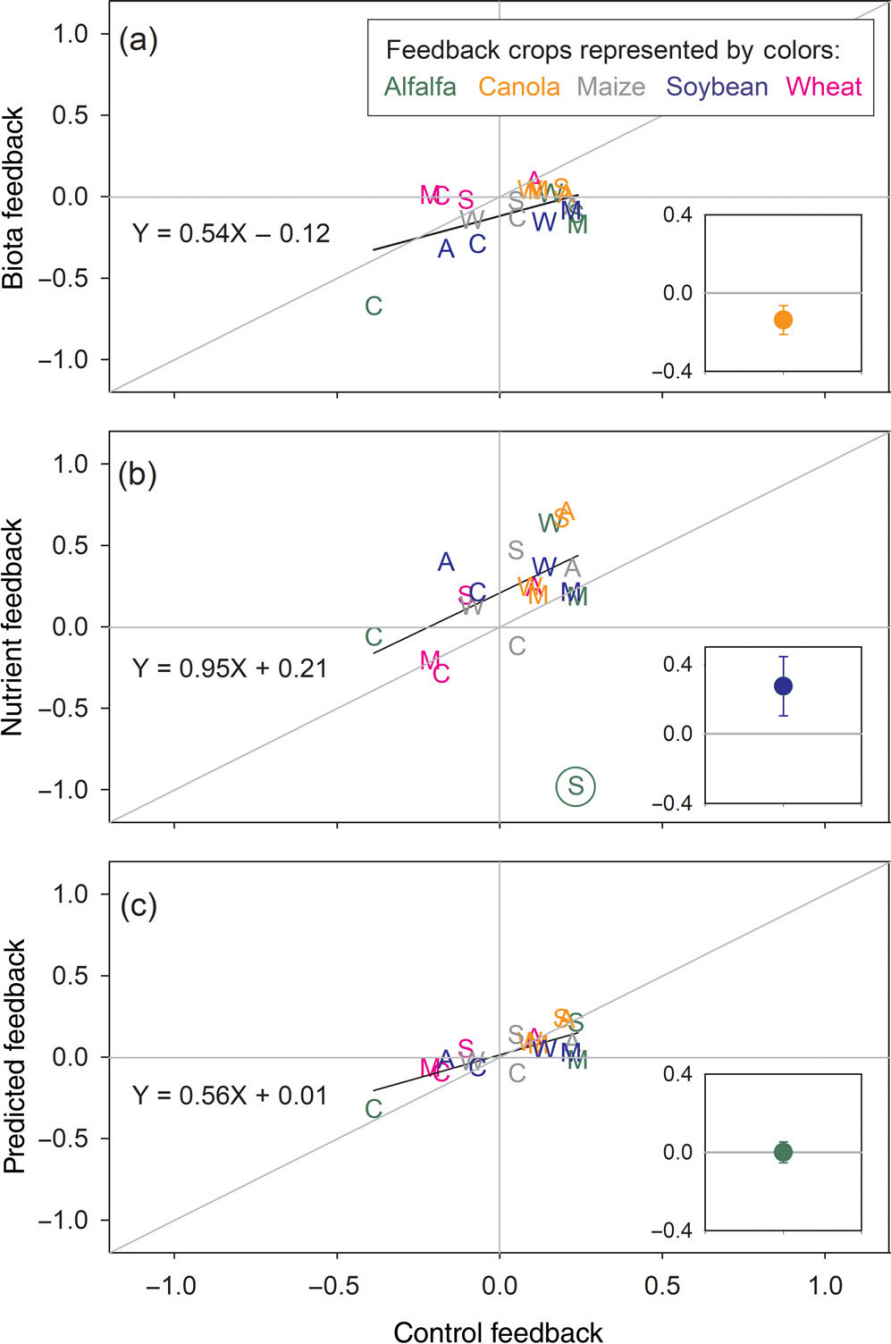
Feedback values of the (a) “biota” and (b) “nutrient” treatments, and (c) predicted values from the model were plotted against the “control” feedback values. The five feedback crops were represented by different colors; alfalfa: green, canola: orange, maize: gray, soybean: blue, and wheat: pink. The training crops were represented by letters: A, C, M, S, and W stand for alfalfa, canola, maize, soybean, and wheat, respectively. Each letter represents an average feedback value (Figure 6). A black regression line resulted from a Pearson correlation analysis. The regression for the control vs. nutrient feedback values (b) resulted from an analysis using a data set without the value of alfalfa grown in soils trained by soybean (circled). Results of the regression analyses are in Table 2. The gray diagonal lines represent 1:1 ratio. Insets show mean differences (i.e., control feedback values subtracted by x‐axis values) and 95% confidence intervals

To test the second hypothesis (control feedback values can be predicted using biotic and nutrient feedback values), we first compared feedback values among the three soil treatments. When feedback values of the biota and control soils were compared, the biota feedback was mostly lower (i.e., less positive or more negative shown below the 1:1 line; *p* < 0.05; Figure 7a inset) than the control; the only exception was wheat (Figure 7a). There was a significant positive correlation between biota and control feedback values (*p* = 0.012; Model 1a in Table 2, Figure 7a). In contrast, when feedback values of the nutrient and control treatments were compared, the effect of the nutrient treatment was mostly higher (i.e., more positive or less negative shown above the 1:1 line; *p* < 0.05; Figure 7b inset); the obvious exception was alfalfa grown in soils trained by soybean (Figure 7b) which can be considered as an outlier (*p* = 0.005 via a Grubbs’ test). There was no significant correlation in feedback values between the nutrient and control soils when all the data points were used (*p* = 0.308; Model 1b in Table 2, Figure 7b). However, when the outlier (i.e., alfalfa grown in soils trained by soybean) was removed, there was a significant positive correlation (*p* = 0.005, Model 2b in Table 2, Figure 7b). There was no significant correlation between the biota and nutrient feedback values (*p* = 0.42; Appendix S1: Figure S10).

**TABLE 2:** ANOVA results to assess if the “control” feedback values could be predicted by feedback values of the “biota” and “nutrient” soils using the full data set and a reduced data set excluding the outlier (alfalfa grown in soils trained by soybean; Figure 7b)

Data set	Model	Biota	Nutrient	Biota × nutrient	*R* ^2^	AIC
Full	**Model 1a**	*F*_1,18_ = 7.79 ***p* = 0.012**	N/A	N/A	0.30	**−14.99**
	Model 1b	N/A	*F*_1,18_ = 1.10 *p* = 0.308	N/A	0.06	22.19
	Model 1c	*F*_1,17_ = 7.56 ***p* = 0.014**	*F*_1,17_ = 0.48 *p* = 0.499	N/A	0.32	−12.61
	Model 1d	*F*_1,16_ = 7.48 ***p* = 0.015**	*F*_1,16_ = 0.47 *p* = 0.502	*F*_1,16_ = 0.81 *p* = 0.383	0.35	−11.59
Reduced	Model 2a	*F*_1,17_ = 8.28 ***p* = 0.010**	N/A	N/A	0.33	−13.68
	Model 2b	N/A	*F*_1,17_ = 10.34 ***p* = 0.005**	N/A	0.38	1.09
	**Model 2c**	*F*_1,16_ = 12.02 ***p* = 0.003**	*F*_1,16_ = 8.68 ***p* = 0.009**	N/A	0.56	**−20.27**
	Model 2d	*F*_1,15_ = 11.26 ***p* = 0.004**	*F*_1,15_ = 8.14 ***p* = 0.012**	*F*_1,15_ = 0.00 *p* = 0.998	0.56	−18.27

*Note*: Four models were built for each data set: control feedback values were a function of (a) biota, (b) nutrient, (c) biota and nutrient (additive), and (d) biota, nutrient and their interaction (interactive) feedback values. Relative quality of the four models was evaluated via Akaike information criteria (AIC). Bold *p* values indicate statistical significance (*p* < 0.05). A bold AIC value and the associated model indicate the best model for a given data set.

Results of linear fixed‐effect ANOVAs in combination with Akaike information criteria (AIC) were used to test if the control feedback values could be predicted using the feedback values of the biota and nutrient treatments (Table 2). When all the data points were used, the control feedback values were best predicted by the biota feedback values with a relatively low coefficient of determination (*R* ^2^ = 0.30, Model 1a in Table 2). When the outlier (i.e., alfalfa grown in soils trained by soybean) was removed from the data set, the control feedback values were best predicted by an additive model carrying both biota and nutrient feedback values with a higher coefficient of determination (*R* ^2^ = 0.56; Model 2c in Table 2, Figure 7c). The coefficients for the biota and nutrient feedback values were 0.44 and 0.32, respectively in the best model (Model 2c in Table 2). The lack of a biota × nutrient interaction effect (*p* = 0.998) was demonstrated by the result of the interactive model (Model 2d in Table 2).

**FIGURE 8:**
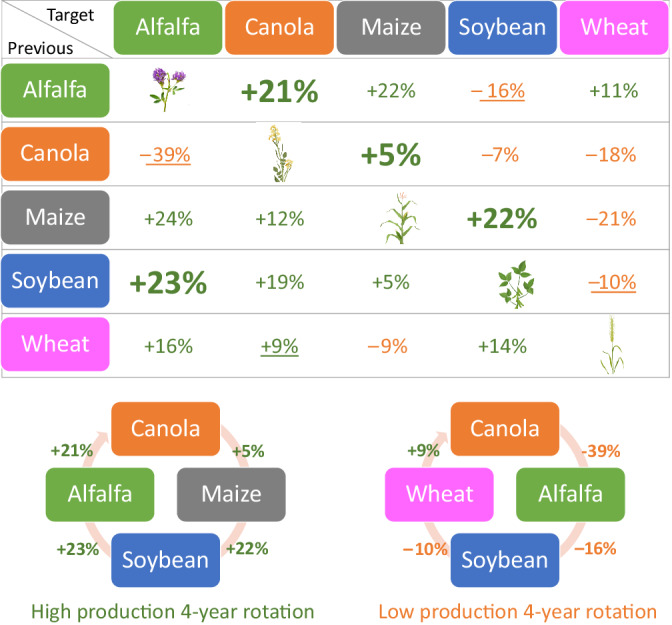
The upper panel shows direction and magnitude of total biomass based on feedback values in the “control” treatment shown in Figure 6: the percentage difference in the growth of a crop after a given different preceding crop (i.e., in rotation) compared to that after the same preceding crop (i.e., as a continuous monoculture) of the control soils. The lower panel shows examples of the high and low production 4‐year rotation scenarios involving canola based on total biomass. Percentages used for the high and low production rotations shown in the lower panel are bolded and underlined in the upper panel, respectively

Using the feedback results in the “control” soils, hypothetical 4‐year crop rotation scenarios starting with canola were constructed to maximize and minimize total biomass (Figure 8), assuming that previous crops only have the greatest legacy effects to the following crops. The high production rotation sequence was canola followed by maize, soybean, and alfalfa; and the low production sequence was canola, followed by alfalfa, soybean, and wheat before going back to canola (Figure 8).

## DISCUSSION

The results of this study indicate that PSF mechanisms can differentially affect the performance of individual crops, demonstrated by varying PSF values across the crop combinations between the training and feedback phases. Consequently, higher crop yields may be obtained through manipulating crop rotation sequences as guided by PSF responses. There was a significant positive correlation in feedback values between the control and biota treatments, and this also appeared to be the case between the control and nutrient treatments when the outlier was removed. This finding demonstrates that soil biota and nutrients were integral parts of soil feedback. Biota and nutrient soil feedback values were mostly below and above the 1:1 line, respectively, when plotted against the control feedback values. The control feedback values were best predicted by an additive model using the biota and nutrient feedback values. Together, these findings suggest that feedbacks resulting from soil biota and nutrients canceled each other out to shape the overall feedbacks (i.e., seen here in control soils). Our result in the control treatment showed that some crop sequences resulted in less biomass production than monocropping. This finding emphasizes that it is critical to assess crop rotation sequences that can maximize crop yields. As such, feedback values obtained from a pot experiment using field soils (i.e., without autoclaving) have the potential to serve as basis to determine rotation sequences that optimize yield via PSF in agricultural fields.

To our knowledge, this is one of the first studies attempting to apply knowledge of PSF, which has been mostly confined to the field of community ecology, to the selection of crop rotation sequences. To that effect, we teased apart the contributions of soil biota and nutrient effects to the overall PSF responses, assuming that crops in the training phase overrode any potential PSF legacies from the previous vegetation in the field across the three treatments. Factors driving PSFs can be categorized into four factors: pathogens, mutualists, nutrients, and secondary chemicals (Smith‐Ramesh & Reynolds, 2017). This study was designed so that the net effect (i.e., the legacies left by training crops) of pathogens and mutualists were accounted for via the biota treatment. In addition, the isolated effects of nutrients, and potentially, secondary chemicals were accounted for by using the nutrient treatment. These major factors can be interdependent of one another (Smith‐Ramesh & Reynolds, 2017) and their interacting effects could have provided complex feedback results difficult to partition. However, our analyses using the fixed‐effect ANOVAs and the AIC model selection process showed that the PSFs in the control treatment were best explained by additive effects from the biota and nutrient feedbacks, indicating that the interdependence between soil biota and nutrients was undetected in our experiment.

The two legume crops, alfalfa and soybean, contributed to the overall less‐negative/more‐positive feedback trend in the nutrient treatment. It is well known that incorporating N_2_ fixing crops in rotations replenishes N in soils, boosting the yields of subsequent crops (Fustec et al., 2010), which is reinforced by our results. Roots of alfalfa and soybean plants can be associated with N_2_‐fixing bacteria (Hayat et al., 2010) and increase N availability to subsequent crops (Crews & Peoples, 2005). Our result highlights the critical role of legumes in crop rotations for sustainable agriculture: legumes can replenish N in soils, which would in turn help reduce the use of N fertilizer and associated energy (Stagnari et al., 2017). Alfalfa and soybean plants likely increased soil N availability via N rhizodeposition (Fustec et al., 2010) in the nutrient treatment, even though the autoclaving process before the training phase eliminated soil microbes, including symbiotic N_2_‐fixing bacteria. This is supported by the observation that all the crops in the feedback phase tended to have greater shoot N contents in soils trained by the leguminous than by the nonleguminous crops. In addition, some symbiotic N_2_‐fixing bacteria could have been introduced via airborne cross contamination from infected alfalfa and soybean roots during the training phase (Abd Aziz et al., 2018), which might have helped add even more N to the soils trained by alfalfa and soybean. Consistent with this assertion, we found nodules in roots of some alfalfa and soybean plants in the nutrient treatment at the end of the feedback phase (Appendix S1: Figure S11).

Alfalfa grown in soils trained by soybean in the nutrient treatment had the most negative feedback across the treatments in this study. This negative feedback was so striking that we considered it an outlier in the model selection analyses. Although we are unable to pinpoint the exact causes for such extreme negative feedback, we note that consecutive cultivation of phylogenetically close crops tends to negatively affect crop yields (Miller & Menalled, 2015). As such, because alfalfa and soybean are more closely related to each other compared to the other crops in this study (Raju et al., 2018), this could offer a plausible explanation for our findings. Moreover, this is further supported by the negative feedback for alfalfa grown after soybean in the biota treatment. Although niche overlap and pathogen overload might have played a role in these treatments, the feedback value for this same crop sequence in the control treatment was positive. This indicates, on one hand, that phylogenetic relatedness and the associated niche overlap alone do not serve as a viable explanation and, on the other hand, that possible biotic filtering might have played a role in the biota treatment. A similar extreme negative feedback was not, however, found in soybean grown after alfalfa in the nutrient treatment. In fact, this feedback turned out positive, suggesting that soybean required greater quantities of limiting nutrients than alfalfa as supported by the greater shoot biomass of soybean (average 1.6 g ± 0.2 SE in dry weight) than alfalfa (average 0.3 ± 0.1 SE) harvested in the training phase. All things considered, we can hypothesize that, under some circumstances, the legacy of soybean to alfalfa, but not vice versa, can consist of both a soil depleted of essential nutrients and rich in biotic antagonists on that crop.

Feedback in the biota treatment tended to be more negative/less positive compared to the control treatment. This indicates that an overall negative effect caused by antagonistic soil biota, such as pathogens, was dominant over a positive effect from beneficial soil biota. On the other hand, feedback in the nutrient treatment tended to be more positive/less negative compared to the control treatment. These observations suggest that potential negative effects in crop rotation via PSF caused by soil biota can be alleviated by nutrient optimization via niche complementarity. These results are consistent with a study by Menalled et al. (2020a) who reported greater plant biomass in sterilized than in inoculated soils across diverse types of soil and plants. In the experiment by Menalled et al. (2020a), the sterilized and inoculated soils had nutrient and biota legacies, respectively, consistent with those in this study. One possible explanation for the negative/less positive feedback in the biota than control treatment is that beneficial effects of symbiotic AMF were suppressed because of high nutrient availability caused by the autoclaving process. Autoclaving often increases availability of soil nutrients, such as N and P (Serrasolsas & Khanna, 1995a, 1995b) because of the thermal breakdown of organic matter and nutrient release from dead microbes. The greater biomass of the nonleguminous crops in the biota and nutrient treatments relative to that in the control treatment where the soil was not autoclaved, supports this hypothesis. In soils rich in nutrients, particularly P, beneficial effects of AMF for their host plants tend to decrease (Hodge et al., 2010; Hoeksema et al., 2010; Johnson, 2010; Johnson et al., 1997). Thus, it is possible that this was the mechanism for the potential reduction of beneficial effects by AMF in the biota treatment. Future assessments of feedback for the selection of crop rotation sequences may benefit from using gamma radiation for soil sterilization, as it has been shown to have less impact on soil characteristics compared to autoclaving (Salonius et al., 1967).

Canola, compared to other crops, generally had a negative effect on subsequent crop biomass via feedback mechanisms, and this was observed across all three soil treatments. This is consistent with reports that canola tends to suppress yields of subsequent crops, such as wheat (Hansen et al., 2019) and maize (Arihara & Karasawa, 2000; Gavito & Miller, 1998; Koide & Peoples, 2012), though such growth suppression is not universal (Robertson et al., 2009; Smith et al., 2004). One plausible explanation for these observations may be associated with allelopathic compounds produced by canola, which can directly influence the performance of crops grown in soils after canola (Yasumoto et al., 2011). This mechanism may explain the substantially smaller biomass of nonlegume crops (by 52.9%, 67.2%, and 63.8% for canola, maize, and wheat, respectively) in the nutrient treatment compared to that in the biota treatment, which by design would not contain allelopathic compounds. However, such biomass differences were not found for the legumes, supporting the hypothesis that the allelopathic effect of canola may be context dependent (Walsh et al., 2014). Autotoxicity of canola (Yasumoto et al., 2011) was apparent in that canola did no grow well in self‐cultivated soils. However, this was not the case for autotoxicity of alfalfa (Chon et al., 2002; Miller et al., 1988) and wheat (Lodhi et al., 1987; Wu et al., 2001, 2007). Thus, degrees of autotoxicity appeared to be crop specific, but it is possible that the observed varying autotoxicity was due to the specific cultivars used in this study (Chung & Miller, 1995; Wu et al., 1999, 2000).

Because canola does not establish associations with AMF, it may negatively impact subsequent crops by reducing AM fungal abundance in soil (Arihara & Karasawa, 2000; Gavito & Miller, 1998; Hansen et al., 2019; Koide & Peoples, 2012). Indeed, arbuscular colonization rates of wheat were substantially lower when wheat was grown in soils after canola compared to other crops in the biota and control treatments (6.1%–9.4% and 6.9%–11.8%, respectively). However, such negative effect on AM root colonization was not apparent in alfalfa, maize, or soybean, suggesting that crop identity may be an important factor in this context (Robertson et al., 2009). Furthermore, canola may not have negative effects on symbiotic N_2_‐fixing bacteria (Pellerin et al., 2007). In some cases, canola reduces antagonistic soil microbes, thereby benefiting subsequent crops such as potato (Larkin et al., 2010) and tobacco (Fang et al., 2016). For instance, take‐all fungus associated with wheat has been shown to be suppressed by canola extracts under laboratory conditions (Angus et al., 1994). However, this beneficial effect may not be apparent in the field (Smith et al., 2004).

Mycorrhizal root colonization, assessed via arbuscular and vesicular colonization, was not a good predictor of plant biomass in either the control or biota treatments. In general, mycorrhizal colonization is positively correlated with biomass, primarily because of the stimulated P uptake by AMF (Treseder, 2013). However, this relationship depends, in part, on the mycorrhizal taxa present (Treseder, 2013). It is possible that the colonization rates per se may not be as important as the community structure of AMF in this study (Gustafson & Casper, 2006; Koch et al., 2017). The only notable relationship was found in soybean grown in the biota treatment, where arbuscular and vesicular colonization rates were positively and negatively correlated with plant biomass, respectively. Arbuscules are a primary organ where nutrients and carbon are exchanged between the symbionts (Parniske, 2008). Therefore, the high arbuscular colonization may be indicative of greater resource exchange between symbionts (Fitter, 2006; Mäder et al., 2000). Being a legume, it is likely that soybean was not limited by N but P in the biota treatment and, as a result, was more prone to benefiting from AM arbuscular colonization. The negative correlation between vesicular colonization and plant biomass may be explained by the nature of vesicles as a storage organ (Parniske, 2008), which require extra energy for their formation when nutrients such as P are limiting (IJdo et al., 2010; Johnson, 1993).

The frequency of root lesions was not an important predictor of plant biomass. The only significant correlation between root lesions and plant biomass was found for alfalfa in the control treatment. However, the direction of this relationship was positive, which is contrary to the assumption that root lesions are a symptom of disease or herbivory. Although root lesion frequency has been used in this context (Dukes et al., 2019; Maron et al., 2014; Martínez‐García et al., 2015; Schnitzer et al., 2011), the data are inconsistent. Some pathogens may not cause root lesions, resulting in underestimation of disease effects (Dukes et al., 2019).

In this study, we focus on the effect of PSFs on the biomass of five crops postgermination. However, seed germination (Li & Romane, 1997) and survival (Packer & Clay, 2000) can be also influenced by PSFs. For instance, Menalled et al. (2020b) reported that germination rates of safflower, clover, and wheat were not affected by farming practices (tilling and organic farming), but by combinations of conditioning and feedback crops. Benitez et al. (2017) also reported that maize seed germination success was greater in soils conditioned by sunflower than other crops including maize, pea, and soybean. One reason why we germinated seeds on sterilized vermiculite was to grow the five different crops in the total of 600 pots in the same time frame in the feedback phase. Germination time and rates varied among the five crops, thus we coordinated seed stratification and germination timings by considering the differences so that all the 600 individual plants could grow simultaneously in the same greenhouse conditions. Effects of PSFs on crop seed germination are, however, important and should thus be explored in future studies.

We used biomass to assess PSFs for the reciprocal combinations of the five crops assuming that biomass of plants grown in pots for 10 weeks in a greenhouse reflect potential crop yields. This assumption might not hold true in the field given that grain yields may not correlate with total biomass at any phenological stage and this relationship can vary among the five crops. Although positive correlations between total biomass and grain yield were reported for wheat (Agegnehu et al., 2014; Mirosavljević et al., 2018), barley (Mirosavljević et al.,^2018^), and millet (Matsuura et al., 2012), we have limited knowledge about the biomass‐yield correlations for all the crops used in this study. In addition, actual durations between germination and harvest in the field can be longer than 10 weeks. Thus, future studies need to investigate the biomass‐yield/fitness relationship, and we propose that a field‐based study in combination with a greenhouse experiment similar to this study is the next step to assess importance of PSFs on yields in crop rotations.

The mean feedback values in the control treatment varied from −0.39 (canola after alfalfa) to 0.24 (maize after alfalfa), indicating that crop yields in rotations can be lower than monocropping. This result suggests that it is critical to assess crop rotation sequences to maximize crop yields. To construct the high‐ and low‐production crop rotation sequences based on the control feedback results, we assumed that previous crops leave the greatest legacy effects to the following crops with no carryover effects more than two sequences. To our knowledge, no research has been conducted to test this assumption specifically. However, in a greenhouse pot experiment, Miller and Menalled (2015) demonstrated that identities of plants (seven crop and four weed species) in the conditioning phase determined biomass of each of four crop species in the feedback phase, whereas influence of soil origins (agricultural field or noncultivated pasture) appeared mostly negligible within each of the two soil treatments (autoclaved or inoculated with soil biota). Thus, this result from Miller and Menalled (2015) supports our assumption. Future research is necessary, however, to address carry‐over legacy effects with more than two crop sequences further.

In conclusion, here we provide evidence that PSFs can be useful in agriculture to optimize crop rotation sequences for improved productivity and sustainability. Indeed, our data show that soil feedback can vary widely among crops, indicating that assessing feedback values can have a valuable application in agriculture. Our results from the three soil treatments highlight the individual and combined roles of soil biota and nutrients in PSFs; overall, in rotating crops, effects of antagonistic biota tend to outweigh those of beneficial microbes, whereas subsequent crops benefit from nutrient optimization via niche complementarity. We recommend the implementation of this approach as a first step prior to establishing or adjusting crop rotations in the field. However, future research is needed to ascertain whether the role of soil feedback on crop productivity is as important in the field as it was found to be under controlled environment conditions.

## CONFLICT OF INTEREST

The authors declare no conflict of interest.

## Supporting information


Appendix S1

## Data Availability

Data (Koyama et al., 2021) are available on the Dryad digital repository (https://doi.org/10.5061/dryad.sqv9s4n2r).
