# Supplementary material for: Application of plant–soil feedbacks in the selection of crop rotation sequences
Source: Ecol Appl. 2022 Feb 6;32(2):e2501. doi: 10.1002/eap.2501 (PMC9286821; doi:10.1002/eap.2501)
Supplement: Supplementary file 1 — Appendix S1 [file EAP-32-0-s001.pdf]

Supporting Information. Koyama, A., T. Dias, and P.M. Antunes. 2021. Application of plant-soil feedbacks in the selection of crop rotation sequences. Ecological Applications.

Appendix S1

Table S1. Results of fixed-effect ANOVAs to assess effects of the soil types and previous crops on four plant characteristics for each crop in the feedback phase. AC and VC represent arbuscular and vesicular colonization, respectively.

Response Variable	Feedback Crop	Soil Type	Training Crop	Interaction
Shoot N	Alfalfa (Fig. S2a)	$F_{2,45} = 17.22$<br>***P* < 0.001**	$F_{4,45} = 1.16$<br>$P < 0.341$	$F_{8,45} = 2.59$<br>***P* = 0.020**
	Canola (Fig. S 2b)	$F_{2,45} = 35.56$<br>***P* < 0.001**	$F_{4,45} = 3.98$<br>***P* = 0.008**	$F_{8,45} = 5.75$<br>***P* < 0.001**
	Maize (Fig. S 2c)	$F_{2,45} = 22.07$<br>***P* < 0.001**	$F_{4,45} = 11.32$<br>***P* < 0.001**	$F_{8,45} = 5.92$<br>***P* < 0.001**
	Soybean (Fig. S 2d)	$F_{2,45} = 20.16$<br>***P* < 0.001**	$F_{4,45} = 1.55$<br>$P = 0.204$	$F_{8,45} = 1.03$<br>$P = 0.425$
	Wheat (Fig. S 2e)	$F_{2,45} = 72.8$<br>***P* < 0.001**	$F_{4,45} = 2.74$<br>***P* = 0.040**	$F_{8,45} = 5.05$<br>***P* < 0.001**
Root Lesion	Alfalfa (Fig. S3a)	$F_{2,75} = 36.28$<br>***P* < 0.001**	$F_{4,75} = 1.33$<br>$P = 0.266$	$F_{8,75} = 1.08$<br>$P = 0.385$
	Canola (Fig. S3b)	$F_{2,75} = 55.99$<br>***P* < 0.001**	$F_{4,75} = 1.16$<br>$P = 0.334$	$F_{8,75} = 0.95$<br>$P = 0.482$
	Maize (Fig. S3c)	$F_{2,75} = 84.49$<br>***P* < 0.001**	$F_{4,75} = 0.91$<br>$P = 0.463$	$F_{8,75} = 0.69$<br>$P = 0.699$
	Soybean (Fig. S3d)	$F_{2,75} = 41.6$<br>***P* < 0.001**	$F_{4,75} = 0.71$<br>$P = 0.587$	$F_{8,75} = 0.65$<br>$P = 0.731$
	Wheat (Fig. S3e)	$F_{2,105} = 67.8$<br>***P* < 0.001**	$F_{4,105} = 0.52$<br>$P = 0.723$	$F_{8,105} = 1.16$<br>$P = 0.337$
AC Colonization	Alfalfa (Fig. S4a)	$F_{2,75} = 3480.97$<br>***P* < 0.001**	$F_{4,75} = 0.56$<br>$P = 0.692$	$F_{8,75} = 0.64$<br>$P = 0.738$
	Maize (Fig. S4b)	$F_{2,75} = 17509.22$<br>***P* < 0.001**	$F_{4,75} = 1.46$<br>$P = 0.223$	$F_{8,75} = 1.25$<br>$P = 0.281$
	Soybean (Fig. S4c)	$F_{2,75} = 3261.33$<br>***P* < 0.001**	$F_{4,75} = 0.94$<br>$P = 0.443$	$F_{8,75} = 1.13$<br>$P < 0.355$
	Wheat (Fig. S4d)	$F_{2,75} = 75.44$<br>***P* < 0.001**	$F_{4,75} = 9.65$<br>***P* < 0.001**	$F_{8,75} = 2.83$<br>***P* = 0.008**
VC Colonization	Alfalfa (Fig. S 5a)	$F_{2,75} = 9409.87$<br>***P* < 0.001**	$F_{4,75} = 1.74$<br>$P = 0.151$	$F_{8,75} = 2.48$<br>***P* = 0.019**
	Maize (Fig. S 5b)	$F_{2,75} = 7057.48$<br>***P* < 0.001**	$F_{4,75} = 0.45$<br>$P = 0.77$	$F_{8,75} = 0.44$<br>$P = 0.891$
	Soybean (Fig. S 5c)	$F_{2,75} = 9793.67$<br>***P* < 0.001**	$F_{4,75} = 1.76$<br>$P = 0.146$	$F_{8,75} = 1.74$<br>***P* = 0.103**
	Wheat (Fig. S 5d)	$F_{2,75} = 13350.86$<br>***P* < 0.001**	$F_{4,75} = 9.30$<br>***P* < 0.001**	$F_{8,75} = 3.30$<br>***P* = 0.003**

Table S2. Results of fixed-effect ANOVAs to assess effects of the soil types and previous crops on hyphal colonization rates of each crop in the feedback phase (Fig. S7).

Feedback Phase Crop	Soil Type	Training Crop	Interaction
Alfalfa (Fig. S6a)	$F_{2,75} = 61.35$<br>**$P < 0.001$**	$F_{4,75} = 0.40$<br>$P = 0.810$	$F_{8,75} = 0.70$<br>$P = 0.688$
Canola (Fig. S6b)	$F_{2,75} = 0.07$<br>$P < 0.929$	$F_{4,75} = 0.16$<br>$P = 0.958$	$F_{8,75} = 0.6$<br>$P = 0.774$
Maize (Fig. S6c)	$F_{2,75} = 87.94$<br>**$P < 0.001$**	$F_{4,75} = 2.01$<br>$P = 0.102$	$F_{8,75} = 0.46$<br>$P = 0.879$
Soybean (Fig. S6d)	$F_{2,75} = 39.98$<br>**$P < 0.001$**	$F_{4,75} = 0.62$<br>$P = 0.652$	$F_{8,75} = 0.46$<br>$P = 0.878$
Wheat (Fig. S6e)	$F_{2,75} = 110.69$<br>**$P < 0.001$**	$F_{4,75} = 0.5$<br>$P = 0.735$	$F_{8,75} = 2.18$<br>**$P = 0.039$**

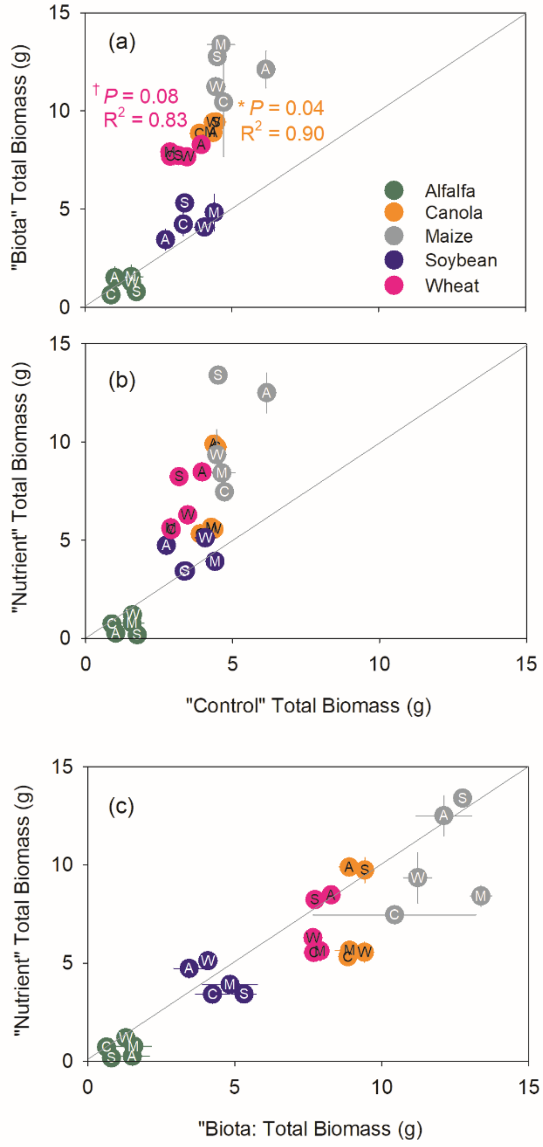

Fig. S1. Relationship between total biomass in ‘control’ and ‘biota’ treatments (a), ‘control’ and ‘nutrient’ treatments (b), and ‘biota’ and ‘nutrient’ treatments (c). Different colors of the circles represent crops in the feedback phase, and letters represent the crops in the training phase: A, C, M, S and W stand for alfalfa, canola, maize, soybean and wheat, respectively. Each symbol and associated error bar represent an average and standard error, respectively (N = 8). The gray diagonal lines represent 1:1 ratio. P values and R^2 show results of Pearson correlation analyses for corresponding crops in the same colors, and associated symbols indicate statistical significance; **: ≤ 0.01, * †: ≤ 0.10. Crops in each panel without P values or R^2 had no significant correlations between X-axis values and total biomass.

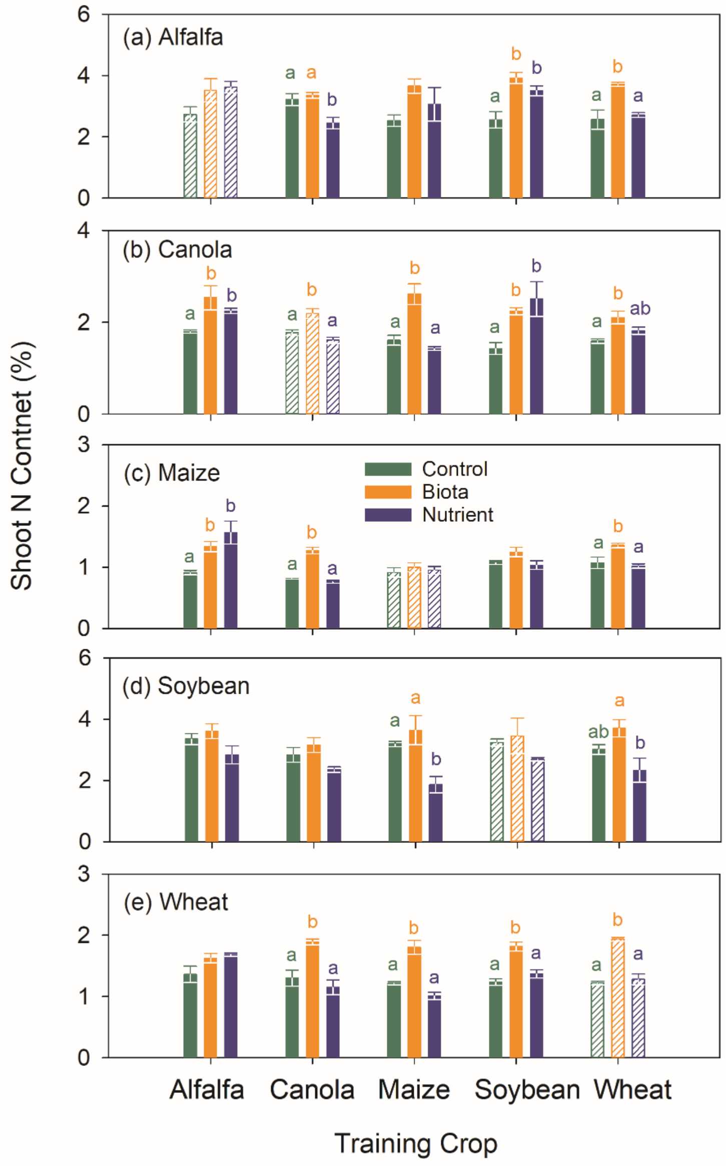

Fig. S2. Shoot N content of the five crops at the end of the feedback phase in the three treatments, 'control', 'biota' and 'nutrient', represented by green, orange, and blue, respectively. The hatched bars represent plants grown in continuous monoculture after the training phase (Fig. 1). Error bars represent standard deviations. Each bar and associated error bar represent an average and standard error, respectively ($N = 8$). Bars topped by different letters among the three soil treatments for each training x feedback crop combination indicate significant difference at $P \leq 0.05$ by a Tukey multiple comparison test. Bars without letters indicate no significant difference among the three soil treatments.

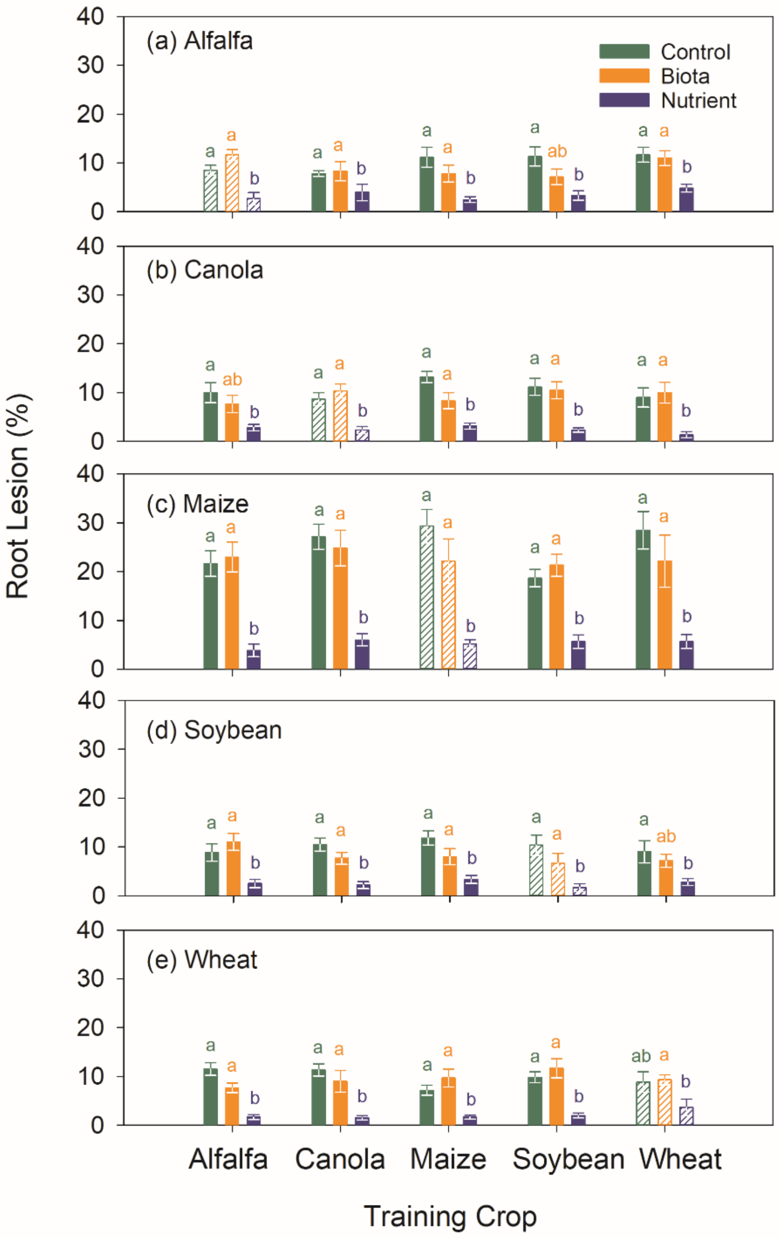

Fig. S3. Root lesion frequency of the five crops at the end of the feedback phase in the three treatments, ‘control’, ‘biota’ and ‘nutrient’, represented by green, orange, and blue, respectively. The hatched bars represent plants grown in continuous monoculture after the training phase (Fig. 1). Each bar and associated error bar represent an average and standard error, respectively (N = 8). Bars topped by different letters among the three soil treatments for each training x feedback crop combination indicate significant difference at $P \leq 0.05$ by a Tukey multiple comparison test.

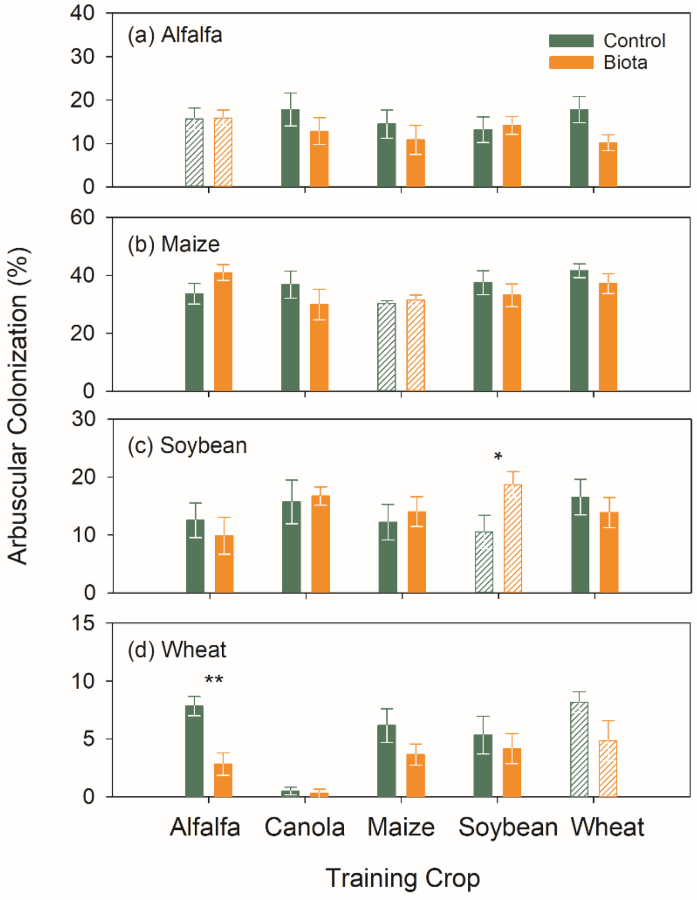

Fig. S4. Arbuscular colonization rates of the five crops at the end of the feedback phase in the two treatments, ‘control’ and ‘biota’, represented by green and orange, respectively. The hatched bars represent plants grown in continuous monoculture after training phase (Fig. 1). No arbuscules were found in non-mycorrhizal canola or in any crops in the ‘nutrient’ treatment. Each bar and associated error bar represent an average and standard error, respectively ($N = 8$). Asterisks above bars show significant differences between the soil treatments for paired samples via Student’s t tests (* $P \leq 0.05$, ** $P \leq 0.01$). Absence of asterisks indicates no significant difference between the two soil treatments.

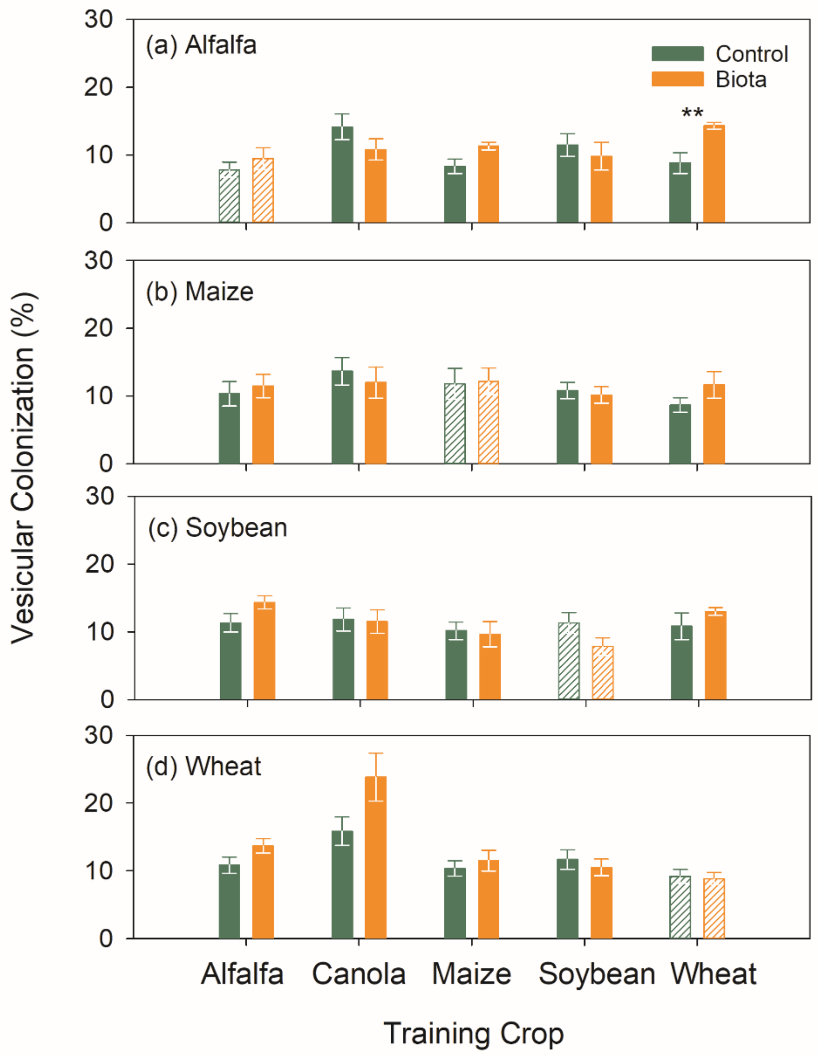

Fig. S5. Vesicular colonization rates of the five crops at the end of the feedback phase in the two treatments, ‘control’ and ‘biota’, represented by green and orange, respectively. The hatched bars represent plants grown in continuous monoculture after the training phase (Fig. 1). No arbuscules were found in non-mycorrhizal canola or in any crops in the ‘nutrient’ treatment. Each bar and associated error bar represent an average and standard error, respectively. Asterisks above bars show significant differences between the soil treatments for paired samples via Student’s t tests (** $P \leq 0.01$). Absence of asterisks indicates no significant difference between the two soil treatments.

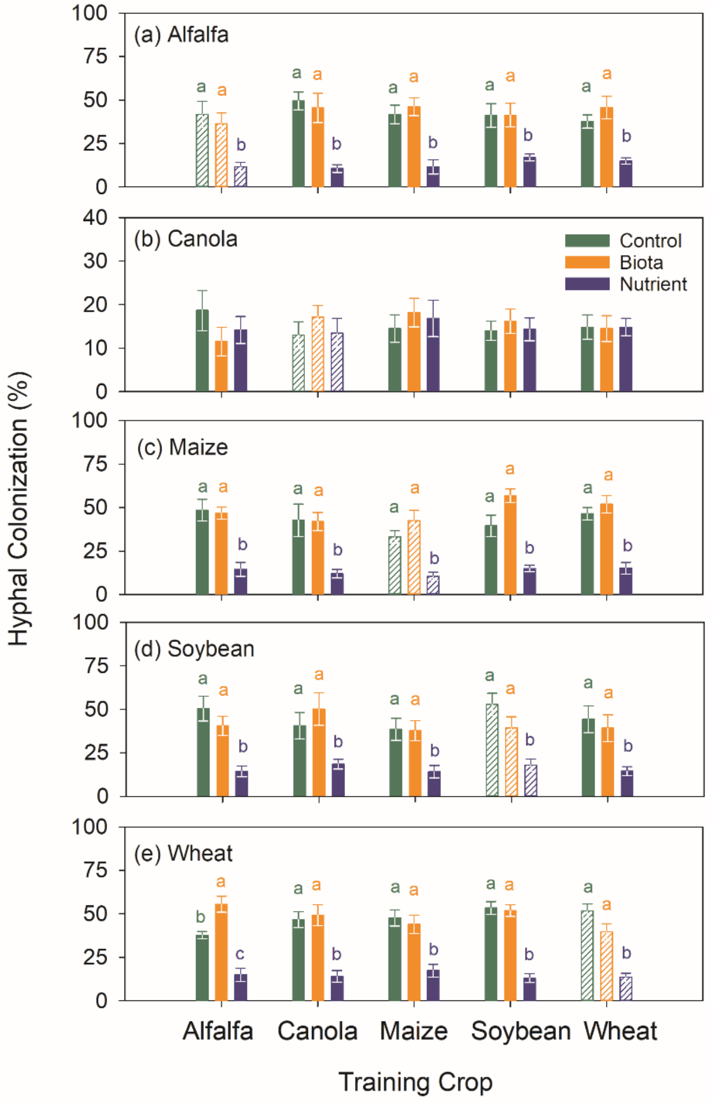

Fig. S6. Hyphal colonization rates of the five crops at the end of the feedback phase in the three treatments, ‘control’, ‘biota’ and ‘nutrient’, represented by green, orange, and blue, respectively. The hatched bars represent plants grown in continuous monoculture after the training phase (Fig. 1). Each bar and associated error bar represent an average and standard error, respectively (N = 8). Bars topped by different letters among the three soil treatments for each training x feedback crop combination indicate significant difference at $P \leq 0.05$ by a Tukey multiple comparison test. Bars without letters indicate no significant difference among the three soil treatments.

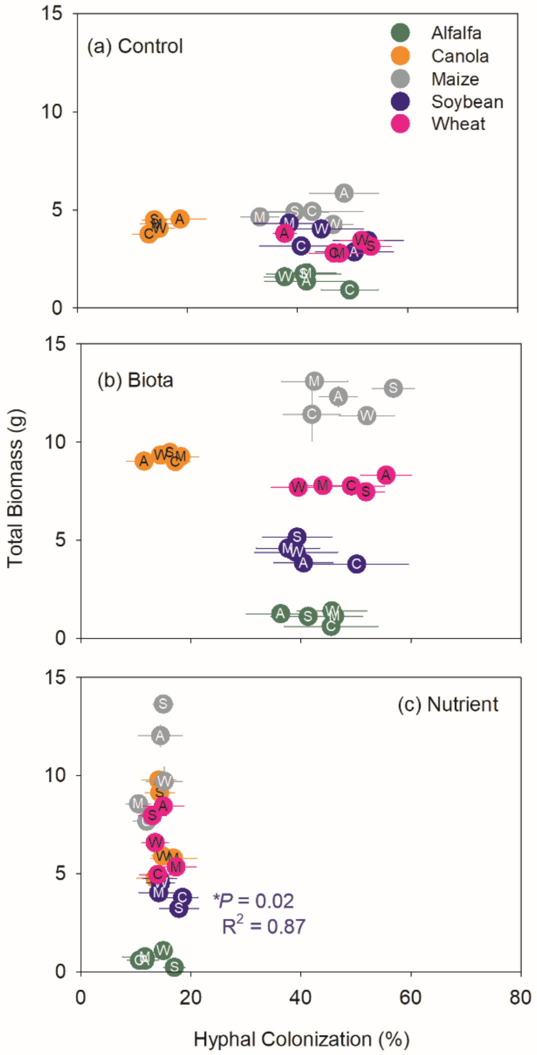

Fig. S7. Relationships between hyphal colonization and total biomass for each treatment (‘control’, ‘biota’ and ‘nutrient’). Different colors of the circles represent crops in the feedback phase, and letters represent crops in the conditioning phase: A, C, M, S and W stand for alfalfa, canola, maize, soybean and wheat, respectively. Each symbol and associated error bars represent an average and standard error, respectively (*N* = 8). Fixed-effect ANOVA results are shown in Table S2.

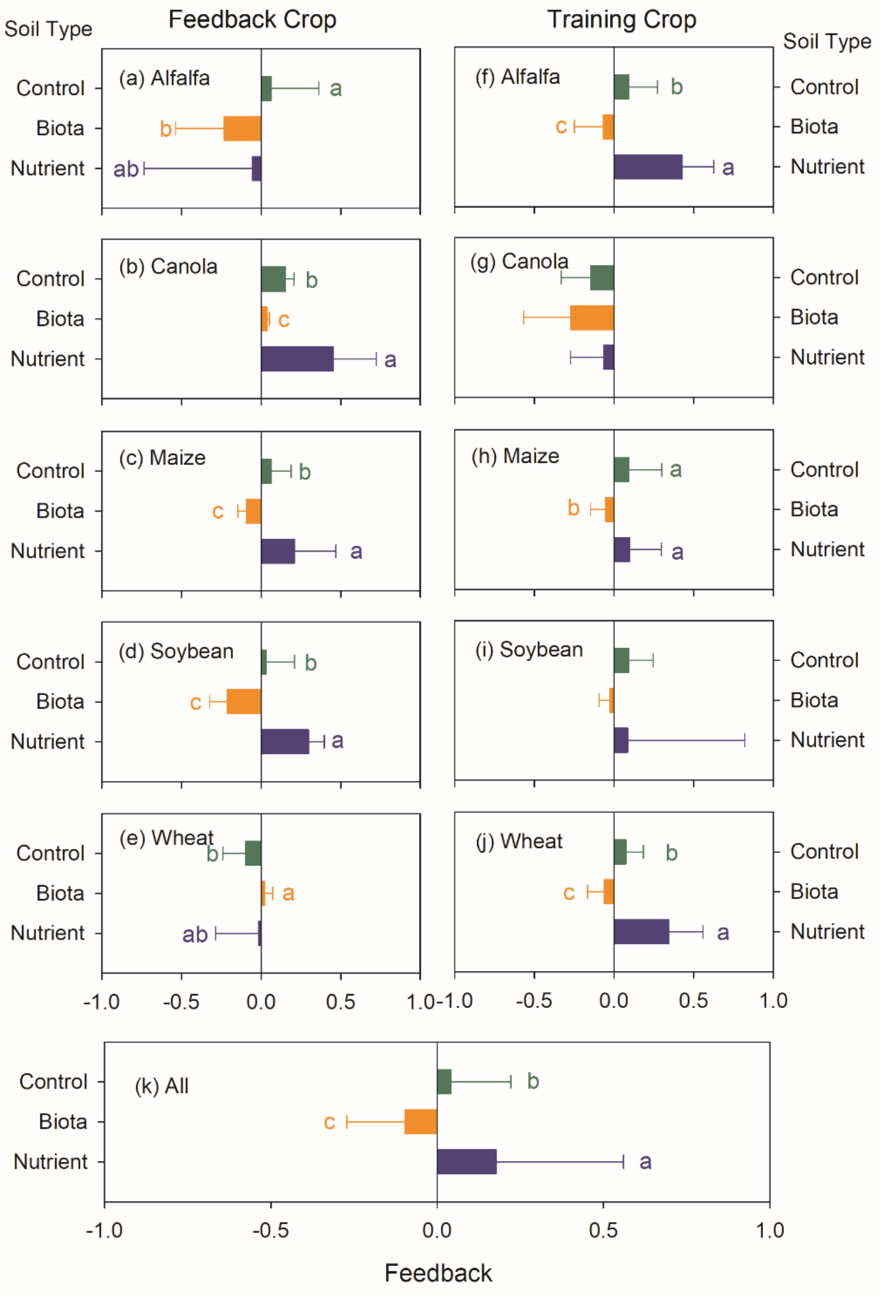

Fig. S8. Feedback values averaged across the feedback (a-e) and training crop (f-j), and all crops (k) for each of the three soil types. Error bars represent standard deviations. Each bar and associated error bar represent an average and standard error, respectively. Bars associated with different letters among the three soil treatments for each crop indicate significant difference at $P \leq 0.05$ by a Tukey multiple comparison test. Bars without letters indicate no significant difference among the three soil treatments.

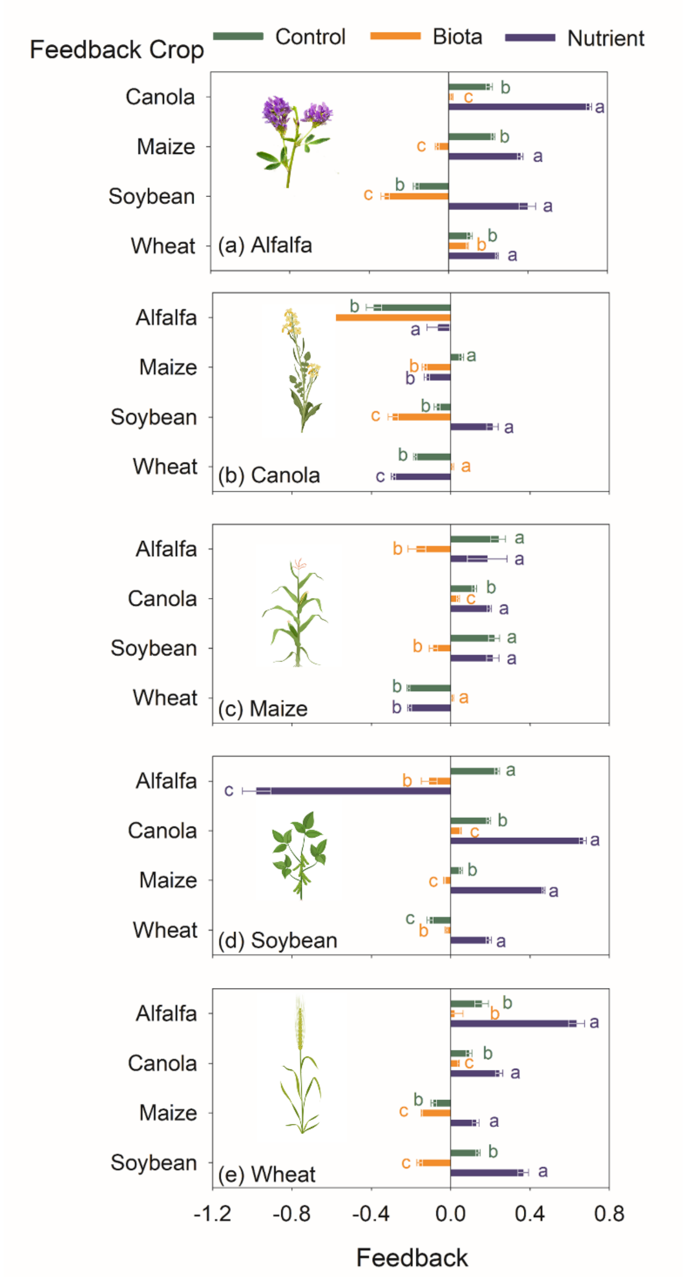

Fig. S9. Soil feedback in three soil types calculated by comparing the total biomass of each crop grown in soils trained by the four other crops against biomass in continuous monoculture after the training phase via bootstrap. The same results shown in Fig. 6 are rearranged for the training crops. Taking alfalfa as an example, a) shows the growth of each of the other crops after alfalfa relative to monocropping of these crops. Each bar and associated error bar represent an average and standard error, respectively. Bars associated with different letters among the three soil treatments for each training x feedback crop combination indicate significant difference at $P \leq 0.05$ by a Tukey multiple comparison test.

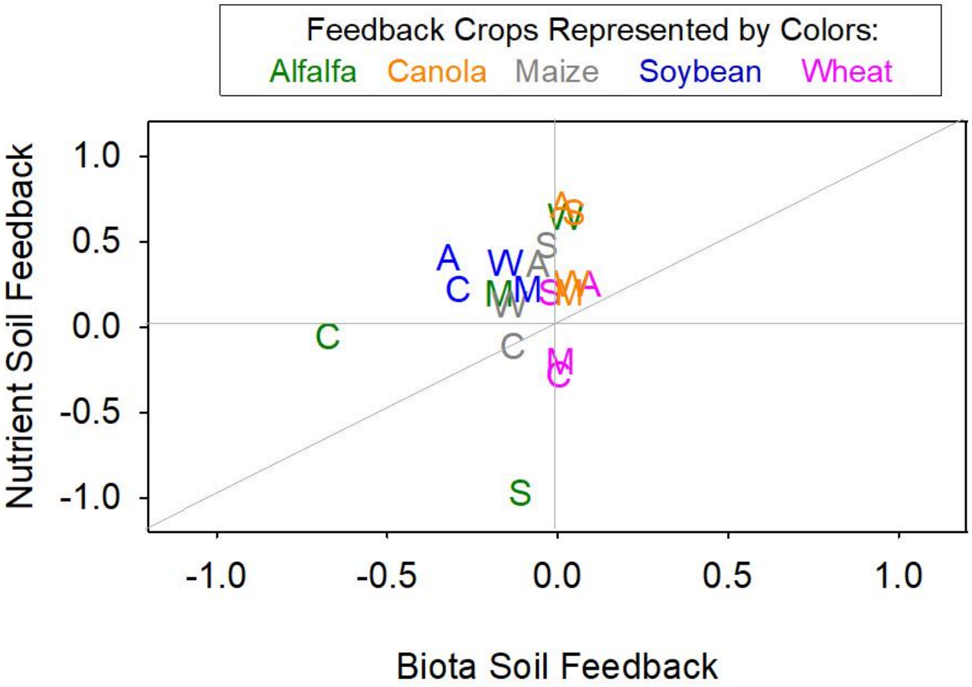

Fig. S10. Feedback values of the ‘biota’ treatment were plotted against those in the ‘nutrient’ treatment. The five feedback crops were represented by different colors; alfalfa: green, canola: orange, maize: gray, soybean: blue, and wheat: pink. The training crops were represented by letters: A, C, M, S and W stand for alfalfa, canola, maize, soybean and wheat, respectively. The gray diagonal lines represent 1:1 ratio.

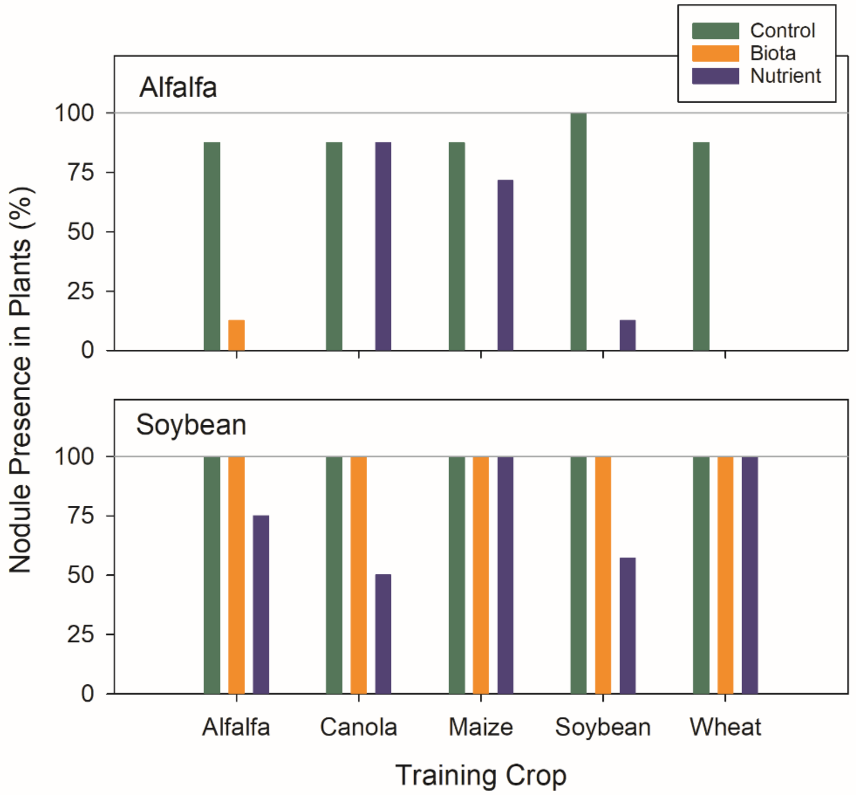

Fig. S11. Frequency of alfalfa and soybean plants in which presence of nodules was confirmed in roots (*N* = 8). No bar indicates that nodules were not found in roots.
